# Psychiatric morbidity and suicidal behaviour in low- and middle-income countries: A systematic review and meta-analysis

**DOI:** 10.1371/journal.pmed.1002905

**Published:** 2019-10-09

**Authors:** Duleeka Knipe, A. Jess Williams, Stephanie Hannam-Swain, Stephanie Upton, Katherine Brown, Piumee Bandara, Shu-Sen Chang, Nav Kapur

**Affiliations:** 1 Population Health Sciences, Bristol Medical School, University of Bristol, United Kingdom; 2 Institute for Mental Health, University of Birmingham, Birmingham, United Kingdom; 3 Sheffield Hallam University, Sheffield, United Kingdom; 4 University Hospital Bristol, Bristol, United Kingdom; 5 University of Nottingham, Nottingham, United Kingdom; 6 Translational Health Research Institute, Western Sydney University, Sydney, Australia; 7 Institute of Health Behaviors and Community Sciences and Department of Public Health, College of Public Health, National Taiwan University, Taipei City, Taiwan; 8 University of Manchester and Greater Manchester Mental Health NHS Foundation Trust, Manchester, United Kingdom; Harvard Medical School, UNITED STATES

## Abstract

**Background:**

Psychiatric disorders are reported to be present in 80% to 90% of suicide deaths in high-income countries (HIC), but this association is less clear in low- and middle-income countries (LMIC). There has been no previous systematic review of this issue in LMIC. The current study aims to estimate the prevalence of psychiatric morbidity in individuals with suicidal behaviour in LMIC.

**Methods and findings:**

PubMed, PsycINFO, and EMBASE searches were conducted to identify quantitative research papers (any language) between 1990 and 2018 from LMIC that reported on the prevalence of psychiatric morbidity in suicidal behaviour. We used meta-analytic techniques to generate pooled estimates for any psychiatric disorder and specific diagnosis based on International classification of disease (ICD-10) criteria. A total of 112 studies (154 papers) from 26 LMIC (India: 25%, China: 15%, and other LMIC: 60%) were identified, including 18 non-English articles. They included 30,030 individuals with nonfatal suicidal behaviour and 4,996 individuals who had died by suicide. Of the 15 studies (5 LMIC) that scored highly on our quality assessment, prevalence estimates for psychiatric disorders ranged between 30% and 80% in suicide deaths and between 3% and 86% in those who engaged in nonfatal suicidal behaviour. There was substantial heterogeneity between study estimates. Fifty-eight percent (95% CI 46%–71%) of those who died by suicide and 45% (95% CI 30%–61%) of those who engaged in nonfatal suicidal behaviour had a psychiatric disorder. The most prevalent disorder in both fatal and nonfatal suicidal behaviour was mood disorder (25% and 21%, respectively). Schizophrenia and related disorders were identified in 8% (4%–12%) of those who died by suicide and 7% (3%–11%) of those who engaged in nonfatal suicidal behaviour. In nonfatal suicidal behaviour, anxiety disorders, and substance misuse were identified in 19% (1%–36%) and 11% (7%–16%) of individuals, respectively. This systematic review was limited by the low number of high-quality studies and restricting our searches to databases that mainly indexed English language journals.

**Conclusions:**

Our findings suggest a possible lower prevalence of psychiatric disorders in suicidal behaviour in LMIC. We found very few high-quality studies and high levels of heterogeneity in pooled estimates of psychiatric disorder, which could reflect differing study methods or real differences. There is a clear need for more robust evidence in order for LMIC to strike the right balance between community-based and mental health focussed interventions.

## Abstract 2

Duleeka Knipe and co-workers study associations between psychiatric disorders and suicidal behaviour in low- and middle-income countries.

## Author summary

**Why was the study done?:**

- Approximately 800,000 people die by suicide each year, with 75% of these deaths occurring in low- and middle-income countries (LMIC).
- Psychiatric disorder is thought to be associated with 80% to 90% of those who engage in suicidal behaviour in high-income countries (HIC), but this association is less clear in LMIC.
- A better understanding is needed of this association in LMIC to ensure effective and appropriate allocation of limited resources.

**What did the researchers do and find?:**

- We searched the existing literature for studies conducted in LMIC that estimated the proportion of individuals who engaged in suicidal behaviour with psychiatric disorders and found 112 studies from 26 countries (mainly from China and India).
- Even in the higher quality studies, there was wide variability in the proportion of psychiatric disorders in fatal (30%–80%) and nonfatal (3%–86%) suicidal behaviour, but on average, 58% of those who died by suicide and 45% of those who engaged in nonfatal suicidal behaviour had a psychiatric disorder.
- Mood disorders were the most prevalent disorder identified in both fatal and nonfatal suicidal behaviour.

**What do these findings mean?:**

- There were considerable differences in the estimates of psychiatric disorder between studies. This may reflect differences in the way studies were carried out or real differences, but there were too few high-quality studies to estimate the prevalence by individual country. More high-quality research is needed.
- Psychiatric disorders may be less common in suicidal behaviour in LMIC than in HIC. Although treating psychiatric illness is important, the treatment and prevention of suicidal behaviour should include a wider range of activities.

## Introduction

At least 800,000 people die by suicide every year, with over three-quarters of these deaths occurring in low- and middle-income countries (LMIC) [1]. Suicidal behaviour is complex with a wide range of contributing factors [2], but it is clear that psychiatric disorders are important in aetiology. The evidence, which largely originates from high-income countries (HIC), estimates that 80% to 90% of people who die by suicide [3–5] and 92% of individuals who attempt suicide [6] have an associated psychiatric disorder. The treatment of these disorders is likely to contribute to suicide prevention efforts.

Psychiatric disorders may play a less prominent role in suicidal behaviour in LMIC, and it has also been argued that the behaviour itself is different in LMIC versus HIC settings [7]. Studies from LMIC provide wide-ranging estimates for the prevalence of psychiatric morbidity in suicidal behaviour, ranging between 10% to 88% [8–10]. A better understanding of the association between psychiatric disorders and suicidal behaviour is important to ensure that the limited resources in LMIC are directed to the appropriate support services.

To our knowledge, there have been no previous meta-analyses of the prevalence of psychiatric morbidity in suicidal behaviour focussed specifically in LMIC. Previous reviews on this topic have focussed on individual regions only [11], excluded non-English language papers [4,6], or included papers that assessed lifetime psychiatric morbidity as opposed to disorders associated with the suicidal act [3]. None of the previous reviews have considered both fatal and nonfatal suicidal behaviour. In this systematic review, we aimed to estimate the prevalence of psychiatric morbidity in individuals who engaged in suicidal behaviour in LMIC by synthesising existing observational evidence. We estimate the prevalence of both overall psychiatric disorders and specific diagnoses based on the International Classification of Diseases (ICD).

## Methods

### Protocol and registration

Our systematic review was conducted following the PRISMA guidelines (S1 PRISMA Checklist) and the protocol registered in advance (PROSPERO 2018 CRD42018087851; see S1 Protocol).

### Eligibility criteria

General population samples from LMIC were included in this review [12]. No age or sex exclusions were made. We included papers that reported on the prevalence of psychiatric morbidity in individuals who engaged in fatal and nonfatal suicidal behaviour. Nonsuicidal self-injury (NSSI) is still a relatively new and understudied area in LMIC, and most studies did not distinguish between NSSI and suicide attempts; for this reason, we did not exclude studies based on suicidal intent nor consider NSSI separately. We included papers that reported on a psychiatric diagnosis based on (i) research diagnostic criteria (i.e., a structured interview), (ii) clinicians’ diagnosis, or (iii) a validated scale with a defined cut-off for case-ness (e.g., Patient Health Questionnaire [PHQ-9]—depression indicated if a score of ≥10 reported). We excluded studies that only reported on either lifetime suicidal behaviour or lifetime psychiatric morbidity because the temporal relationship was then uncertain. We were most interested in the role of psychiatric morbidity as a proximal antecedent to suicide in LMIC rather than an enduring risk factor. An important issue clinically is how many people are suffering with a psychiatric disorder at the time of death. A psychiatric illness diagnosed at some point in the past could have been incidental and not related to the suicidal behaviour. If a study reported on a clinical diagnosis, it was assumed to be the diagnosis at the time of event if not otherwise stated. Studies that reported on a single method of suicidal behaviour were included in the review but were not considered as a high-quality study (see below) because of threats to generalisability. No language restrictions were applied, and papers published between 1 January 1990 and 25 Febuary 2018 were included. We judged studies published prior to 1990 to be of less relevance given the changing global context of suicide [1]. This date also meant that we were not considering diagnoses based on earlier versions of the main classification systems for psychiatric diagnoses (ICD and DSM). Most studies from LMIC that used a structured interview (our gold standard) used a structured clinical interview for DSM (SCID), which was first published in 1990 [13]. If a single study was reported in multiple reports, the report with the most comprehensive data and complete case series was used to extract data.

### Information sources

We searched Medline, PsycINFO, and EMBASE using a combination of key search terms relating to our 3 primary concepts: (i) psychiatric morbidity, (ii) suicidal behaviour (fatal and nonfatal), and (iii) LMIC. The search terms used in Medline are shown in S1 Appendix. Reference searches were conducted on all included papers and relevant reviews.

### Study selection

Titles and abstracts were screened by a single reviewer and a random sample of 30% of articles were independently screened by a second reviewer. The interrater reliability between the reviewers was good (Kappa = 0.75; 95% CI 0.69–0.80) with the initial reviewer being more inclusive. Full-texts were screened independently by 2 members of the research team, and a third reviewer resolved any disagreements; the third reviewer also reviewed all excluded full-text papers. All screening was done using the web application Rayyan [14]. All non-English language papers were screened and data extracted with the help of a native speaker.

### Data extraction

Using a structured data extraction proforma 2 review authors independently extracted data on study design, participants, exposure, and outcome details. When papers presented prevalence estimates for both fatal and nonfatal suicidal behaviour separately, each outcome was included as a separate study (e.g., [15]). If a study reported on both fatal and nonfatal suicidal behaviour but did not provide separate estimates of the prevalence of psychiatric morbidity, the majority outcome (fatal/nonfatal) was used to class the study as either being a study of fatal or nonfatal suicidal behaviour [16–18]. We extracted prevalence estimates for each diagnosis reported in a single paper; this therefore means that a single study contributes more than one prevalence estimate in this review.

### Quality assessment

We used a similar concept for assessing data quality as that employed by the Newcastle-Ottawa scale (NOS) for assessing nonrandomised studies [19]. The NOS does not have a scale for assessing case series; therefore we created a quality assessment tool that assessed potential sources of selection bias and exposure ascertainment. Quality was assessed by 2 independent reviewers. A study was classed as being of high quality if it recruited a consecutive series of suicidal behaviour cases with no threats to generalisability and if the psychiatric diagnosis was reached through a structured interview. The scale used is provided as a supplement (S1 Appendix), and studies were dichotomised as those that scored highly on both the selection of cases and the assessment of psychiatric disorder. Studies were discussed by 2 reviewers, and a consensus rating was recorded.

### Analysis

The prevalence of psychiatric morbidity was recorded as the proportion of individuals who engaged in suicidal behaviour with a psychiatric diagnosis. In some studies, the raw numbers of cases with a diagnosis were not presented, but this was back calculated in order to allow inclusion in the evidence synthesis. We pooled prevalence estimates using random effects meta-analysis using Stata version 15; this was done separately for fatal and nonfatal suicidal behaviour. We used the *metaprop* command in Stata and used a continuity correction for study estimates to ensure that studies with prevalence estimates close to 0% or 100% were not excluded. As recommended, we have used the score methods for the estimation of CIs for our binomial data [20]. Pooled estimates were generated using a random effects model using the method of DerSimonian and Laird [21], with the estimate of heterogeneity being taken from the inverse-variance fixed-effect model. We calculated a pooled estimate for each broad ICD-10 (f-code) diagnostic category separately and for the prevalence of any psychiatric disorder (if this was reported). Between study heterogeneity was assessed using *I*^*2*^ [22]. A pooled estimate was only calculated if there were enough studies in each broad ICD-10 code; codes with less than 5 studies were not meta-analysed. We also present the pooled estimates of the high-quality studies and use this to form the basis of our conclusions. Forest plots were used to graphically present the data, and predictive intervals are presented for the primary analyses. We conducted a set of prespecified subanalyses exploring potential sources of heterogeneity by (i) region, (ii) proportion of males (<50% versus ≥50%—to reflect the known sex differences in psychiatric morbidity in the general population), and (iii) subgrouping of f-codes. We did not generate pooled estimates for subgroups in which there were fewer than 5 studies. We also did not pool the estimates for the f-code subgroup of unspecific mental disorders, because the disorders included in this category are likely to be highly heterogenous. As a post hoc subanalysis, we explored potential sources of heterogeneity by the ages of individuals included in the studies. We grouped studies into those conducted in young people (≤25 years), working age adults (26–65 years), or older adults (>65 years). Studies that included all ages were grouped with the working age adult studies. We also conducted 2 additional subgroup analyses—by assessment type and method of nonfatal case ascertainment. These additional analyses were post hoc and restricted to the main psychiatric morbidities identified in this review. All data used in this analysis can be found in the supporting information (S1 Data).

### Ethics

Ethical approval was not necessary, because this was the analysis of previously published data

## Results

**Fig 1:**
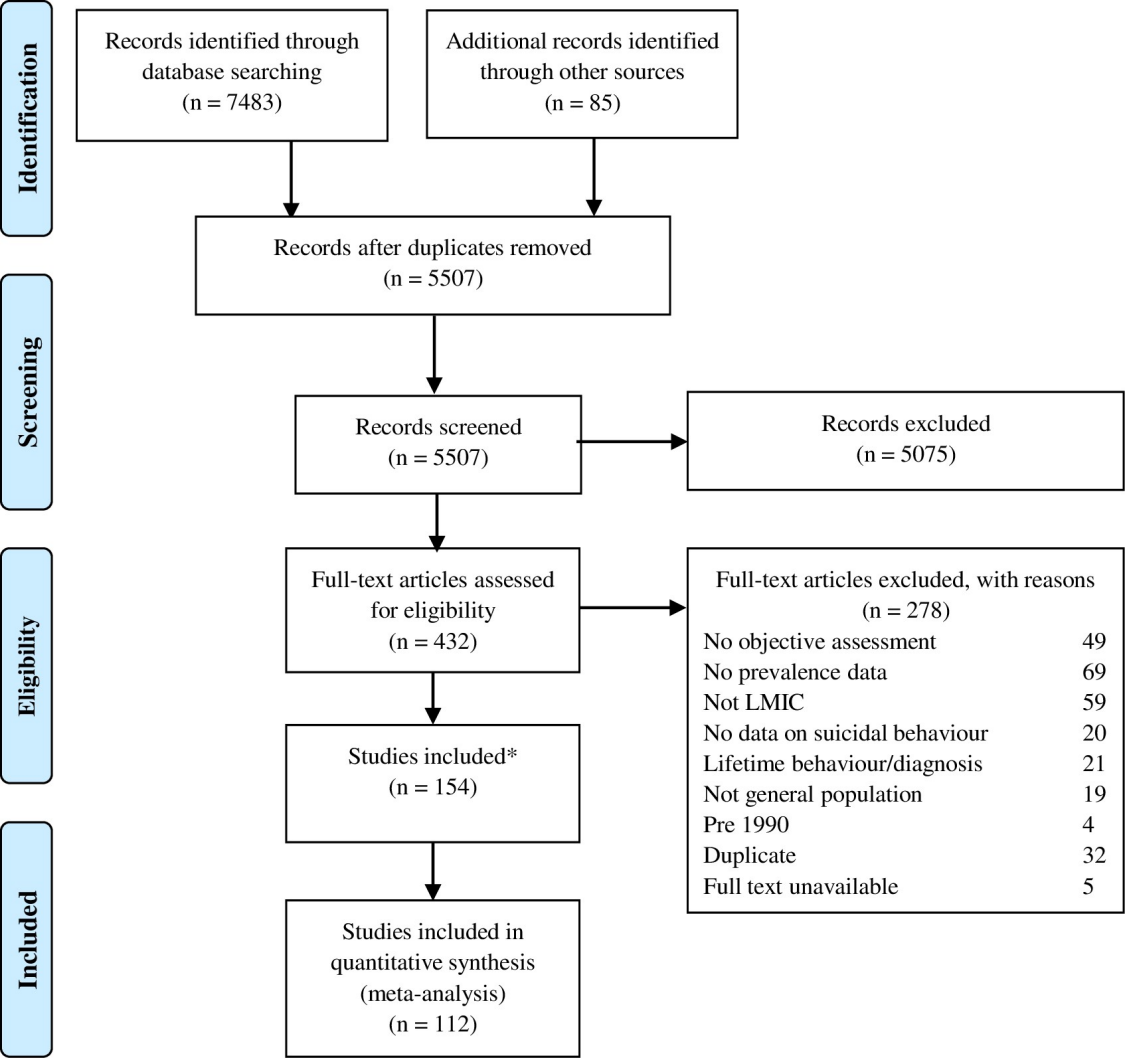
Flowchart of studies included in this review. Includes manuscripts that are multiple reports of the same study sample. Only one instance of each study population is retained and included in the analysis. LMIC, low- and middle-income countries.

Fig 1 shows how we identified 112 studies reported in 154 papers, involving 30,030 nonfatal suicidal behaviour cases (studies *n* = 89) and 4,996 suicide deaths (studies *n* = 23) from 26 LMIC (see Table 1 for study characteristics). This represents 19% of all LMIC and 72% of people who live in LMIC [23]. The countries represented in the suicide studies account for 53% of suicide deaths globally and 70% of LMIC suicide deaths. There were 18 non-English articles included in this review. The countries with the greatest number of studies were from India (*n* = 28; 25%), China (*n* = 17; 15%), and Iran (*n* = 12; 11%). Suicide deaths were primarily identified through coroner’s or police records (*n* = 14) or through active surveillance systems (*n* = 7). Most studies on nonfatal suicidal behaviour (*n* = 81; 91%) were conducted in a hospital setting, with only 8 conducted in a community setting. Most studies either included all methods of suicidal behaviour or did not specify, with only 15 studies reporting on a specific method [17,24–37]. Table 2 and Table 3 summarises the number of studies reporting on each ICD-10 diagnosis category. If a study reported on a diagnosis that was unable to be grouped into a single ICD-10 category, these estimates were excluded (*n* = 12). A total of 17 studies were rated highly in our quality rating (15 of which reported on the overall prevalence of psychiatric disorder and 2 reported on individual diagnoses). These studies were from China, India, Indonesia, Brazil, Ethiopia, and Thailand. These countries represent 52% of global and 68% of LMIC suicide deaths.

**Table 1:** Characteristics of included studies.

Author (year) country	No. cases (% male/age range)	Setting	Assessment type	Outcome	Quality	Single largest method group (%)	Multiple reports
Abbas (2018) Iraq [38]	647 (58%/All ages)	Police	Clinician diagnosis	Fatal suicidal behaviour		Hanging (41%)	
Abeyasinghe (2008) Sri Lanka [39]	372 (79%/≥10)	Multiple sources	Clinician diagnosis	Fatal suicidal behaviour		Pesticide poisoning (70%)	
Ali (2014) Malaysia [40]	328 (74%/All ages)	Coroners	Clinician diagnosis	Fatal suicidal behaviour			
Altindag (2005) Turkey [41]	26 (15%/All ages)	Coroners	Clinician diagnosis	Fatal suicidal behaviour		Hanging (45%)	
Bhise (2016) India [42]	98 (90%/All ages*)	Coroners	Clinician diagnosis	Fatal suicidal behaviour		Pesticide poisoning (68%)	
Hagaman (2017) Nepal [43]	302 (57%/≥18)	Police	PHQ-9	Fatal suicidal behaviour		Hanging (63%)	
Hayati (2004) Malaysia [44]	76 (72%/All ages*)	Coroners	Clinician diagnosis	Fatal suicidal behaviour		Poisoning (39%)	
Jia (2014) China [45]	200 (58%/All ages)	Surveillance	SCID	Fatal suicidal behaviour	High		
Khan (2005) India [46]	50 (0%/15–35)	Coroners	Clinician diagnosis	Fatal suicidal behaviour		Hanging (40%)	
Khan (2008) Pakistan [47]	100 (83%/All ages*)	Police	Clinician diagnosis	Fatal suicidal behaviour		Hanging (40%)	
Khelil (2016) Tunisia [37]	235 (69%/All ages)	Coroners	Clinician diagnosis	Fatal suicidal behaviour		Burning (100%)	
Kurihara (2009) Indonesia [48]	60 (63%/All ages)	Police	SCID	Fatal suicidal behaviour	High	Hanging (68%)	
Maksuti (2012) Albania [49]	254 (64%/All ages)	Coroners	Clinician diagnosis	Fatal suicidal behaviour		Poisoning (39%)	
Manoranjitham (2010) India [50]	100 (59%/All ages*)	Surveillance	SCID	Fatal suicidal behaviour	High		
Palacio (2007) Colombia [51]	108 (81%/All ages*)	Coroners	Clinician diagnosis	Fatal suicidal behaviour		Hanging (47%)	[51]
Samaraweera (2008) Sri Lanka [52]	27 (70%/All ages*)	Coroners	Clinician diagnosis	Fatal suicidal behaviour		Poisoning (70%)	
Taktak (2013) Turkey [53]	124 (67%/All ages)	Coroners	Clinician diagnosis	Fatal suicidal behaviour		Hanging (60%)	
Tong (A) (2018) China [15]	151 (55%/≥18)	Surveillance	SCID	Fatal suicidal behaviour	High	Pesticide poisoning (55%)	[54]
Yang (2005) China [55]	895 (51%/≥10)	Surveillance	SCID	Fatal suicidal behaviour	High	Pesticide poisoning (58%)	[9,56–62]
Zhang (2004) China [63]	66 (73%/All ages)	Surveillance	SCID	Fatal suicidal behaviour		Pesticide poisoning (70%)	[64]
Zhang (2010) China [65]	392 (55%/15–34)	Surveillance	SCID	Fatal suicidal behaviour	High	Pesticide poisoning (73%)	[66–80]
Zhou (2012) China [81]	200 (%/All ages)	Surveillance	SCID	Fatal suicidal behaviour			
Aghanwa (2004) Fiji [82]	128 (31%/All ages)	Hospital	Clinician diagnosis	Nonfatal suicidal behaviour		Drug overdose (51%)	[83]
Ahmadi (2010) Iran [34]	30 (13%/All ages*)	Hospital	Clinician diagnosis	Nonfatal suicidal behaviour		Burning (100%)	
Atay (2012) Turkey [84]	26 (0%/18–65 years)	Community (self-report)	SCID	Nonfatal suicidal behaviour		Drug overdose (73%)	
Ayehu (2017) Ethiopia [85]	96 (42%/≥14)	Hospital	MINI-plus	Nonfatal suicidal behaviour	High	Pesticide poisoning (44%)	
Bansal (1) (2011) India [86]	100 (42%/≥16)	Hospital	Clinician diagnosis	Nonfatal suicidal behaviour		Poisoning (87%)	
Bansal (2) (2011) India [87]	100 (61%/All ages*)	Hospital	Clinician diagnosis	Nonfatal suicidal behaviour		Poisoning (69%)	
Barrimi (2014) Morocco [88]	31 (58%/All ages)	Hospital	Clinician diagnosis	Nonfatal suicidal behaviour		Poisoning (52%)	
Batinic (2012) Serbia [89]	60 (53%/All ages)	Hospital	Clinician diagnosis	Nonfatal suicidal behaviour		Self-cutting (35%)	
Bhattacharjee (2012) India [90]	200 (50%/≥15)	Hospital	Clinician diagnosis	Nonfatal suicidal behaviour			
Bi (2010) China [91]	239 (22%/≥15)	Hospital	SCID	Nonfatal suicidal behaviour	High	Poisoning (92%)	
Bilginer (2017) Turkey [31]	100 (13%/Young people)	Hospital	Clinician diagnosis	Nonfatal suicidal behaviour		Poisoning (100%)	
Borges (2010) Mexico [92]	166 (%/18–65)	Community (self-report)	WHO CIDI	Nonfatal suicidal behaviour			
Caribe (2012) Brazil [29]	110 (46%/–18)	Hospital	MINI	Nonfatal suicidal behaviour		Pesticide poisoning (49%)	
Chandrasekaran (2005) India [93]	341 (45%/18+)	Hospital	MINI	Nonfatal suicidal behaviour		Poisoning (91%)	[94]
Chatterjee (2015) India [95]	100 (34%/All ages*)	Hospital	Clinician diagnosis	Nonfatal suicidal behaviour			
Cherif (2012) Tunisia [96]	30 (%/Adolescents)	Hospital	Clinician diagnosis	Nonfatal suicidal behaviour		Drug overdose (67%)	
Cosar (1997) Turkey [97]	185 (40%/All ages)	Hospital	Clinician diagnosis	Nonfatal suicidal behaviour		Drug overdose (61%)	
de Silva (A) (2000) Sri Lanka [98]	172 (44%/All ages*)	Hospital	Clinician diagnosis	Nonfatal suicidal behaviour		Pesticide poisoning (67%)	
de Silva (B) (2000) Sri Lanka [98]	140 (54%/All ages*)	Hospital	Clinician diagnosis	Nonfatal suicidal behaviour			
Diehl (2009) Brazil [99]	39 (%/All ages)	Hospital	Clinician diagnosis	Nonfatal suicidal behaviour		Drug overdose (48%)	
Farzaneh (2010) Iran [36]	248 (19%/12–18)	Hospital	Clinician diagnosis	Nonfatal suicidal behaviour		Drug overdose (88%)	
Fresan (2015) Mexico [100]	140 (36%/15–60)	Hospital	Clinician diagnosis	Nonfatal suicidal behaviour		Poisoning (44%)	
Galgali (1998) India [35]	308 (51%/All ages*)	Hospital	Clinician diagnosis	Nonfatal suicidal behaviour		Poisoning (100%)	
Gao (2017) China [101]	730 (30%/All ages)	Community (self-report)	BDI	Nonfatal suicidal behaviour			
Ghaleiha (2012) Iran [102]	1,566 (47%/All ages*)	Hospital	Clinician diagnosis	Nonfatal suicidal behaviour		Poisoning (83%)	
Gomes (2009) Brazil [103]	132 (0%/10–49)	Hospital	Clinician diagnosis	Nonfatal suicidal behaviour		Poisoning (95%)	
Grau (2013) Colombia [104]	217 (46%/All ages*)	Hospital	Clinician diagnosis	Nonfatal suicidal behaviour			
Grover (2016) India [105]	109 (48%/<18)	Hospital	Clinician diagnosis and BDI	Nonfatal suicidal behaviour		Pesticide poisoning (52%)	
Haddad (1998) Jordan [33]	20 (20%/All ages*)	Hospital	Clinician diagnosis	Nonfatal suicidal behaviour		Burning (100%)	
Haider (2016) India [106]	100 (28%/All ages*)	Hospital	Clinician diagnosis, BDI	Nonfatal suicidal behaviour		Poisoning (71%)	
Ibiloglu (2016) Turkey [107]	106 (46%/18–55)	Hospital	Clinician diagnosis and SCID-I	Nonfatal suicidal behaviour		Drug overdose (82%)	
Jain (1999) India [108]	56 (59%/≥15)	Hospital	Clinician diagnosis	Nonfatal suicidal behaviour		Pesticide poisoning (57%)	
Jiang (2013) China [109]	297 (26%/All ages)	Hospital	SCID	Nonfatal suicidal behaviour		Pesticide poisoning (81%)	
Kar (2010) India [110]	149 (44%/All ages)	Hospital	Clinician diagnosis	Nonfatal suicidal behaviour		Poisoning (41%)	
Khan (1998) Pakistan [111]	447 (41%/All ages)	Hospital	Clinician diagnosis	Nonfatal suicidal behaviour		Drug overdose (74%)	
Khazaei (2003) Iran [112]	301 (44%/All ages*)	Hospital	Clinician diagnosis	Nonfatal suicidal behaviour			
Kinyanda (2004) Uganda [113]	100 (64%/≥15)	Hospital	Clinician diagnosis	Nonfatal suicidal behaviour		Pesticide poisoning (45%)	
Kulkarni (2013) India [114]	100 (52%/18–55)	Hospital	SCID and International Personality Disorder Examination	Nonfatal suicidal behaviour	High	Pesticide poisoning (71%)	[115,116]
Kumar (1998) India [117]	100 (65%/All ages*)	Hospital	Clinician diagnosis	Nonfatal suicidal behaviour		Pesticide poisoning (53%)	
Kumar (2006) India [118]	203 (51%/16–65)	Hospital	Clinician diagnosis	Nonfatal suicidal behaviour		Poisoning	
Kumar (2015) India [119]	1,159 (50%/All ages)	Hospital	Clinician diagnosis	Nonfatal suicidal behaviour		Poisoning (76%)	
Lari (2007) Iran [25]	89 (21%/All ages)	Hospital	Clinician diagnosis	Nonfatal suicidal behaviour		Burns (100%)	
Latha (1996) India [120]	73 (62%/All ages*)	Hospital	Clinician diagnosis and BDI	Nonfatal suicidal behaviour		Pesticide poisoning (67%)	
Liu (2018) China [121]	409 (32%/15–70)	Hospital	SCID	Nonfatal suicidal behaviour	High		[121–123]
Ma (1999) China [124]	98 (20%/All ages*)	Hospital	Chinese mental health screening scale	Nonfatal suicidal behaviour		Drug overdose (75%)	
Maselko (2008) India [125]	2,318 (0%/18–45)	Community (self-report)	CIS-R	Nonfatal suicidal behaviour			
Mechri (2005) Tunisia [126]	90 (59%/All ages)	Hospital	Clinician diagnosis	Nonfatal suicidal behaviour		Drug overdose	
Mohammadi (2005) Iran [127]	362 (30%/≥18)	Community (self-report)	SADS	Nonfatal suicidal behaviour			
Moosa (2005) South Africa [128]	43 (40%/All ages)	Hospital	Clinician diagnosis	Nonfatal suicidal behaviour		Drug overdose (63%)	
Mugisha (2015) Uganda [129]	17 (%/≥18)	Community (self-report)	MINI-plus	Nonfatal suicidal behaviour			
Muralidhara (2011) India [130]	51 (53%/18–65)	Hospital	SCID	Nonfatal suicidal behaviour		Pesticide poisoning (68%)	
Naidoo (2013) South Africa [131]	688 (25%/≥18)	Hospital	BDI	Nonfatal suicidal behaviour		Poisoning (92%)	
Narang (2000) India [132]	100 (58%/All ages*)	Hospital	Clinician diagnosis	Nonfatal suicidal behaviour		Pesticide poisoning (77%)	
Ndosi (1997) Tanzania [133]	300 (31%/All ages)	Hospital	Clinician diagnosis	Nonfatal suicidal behaviour		Overdoses (91%)	
Ozdel (2009) Turkey [134]	144 (25%/All ages*)	Hospital	SCID	Nonfatal suicidal behaviour		Drug overdose (70%)	
Paholpak (2012) Thailand ** [18]	8,426 (%/All ages)	Hospital	Clinician diagnosis	Nonfatal suicidal behaviour	High	Poisoning (89%)	
Pandey (2013) India [135]	80 (53%/18–60)	Hospital	SCID-I and II	Nonfatal suicidal behaviour			
Parkar (2006) India [136]	196 (53%/≥18)	Hospital	SCID	Nonfatal suicidal behaviour		Pesticide poisoning (74%)	[137]
Pearson (2002) China [138]	147 (0%/15–35)	Hospital	SCID	Nonfatal suicidal behaviour		Pesticide poisoning (88%)	
Pérez-Olmos (2007) Colombia [139]	96 (27%/11–18)	Hospital	Clinician diagnosis	Nonfatal suicidal behaviour		Poisoning (97%)	
Qusar (2009) Bangladesh [140]	44 (41%/All ages*)	Hospital	Clinician diagnosis	Nonfatal suicidal behaviour		Poisoning (95%)	
Rajapakse (2014) Sri Lanka [28]	949 (44%/≥14)	Hospital	GAD-7, PHQ-9, and AUDIT	Nonfatal suicidal behaviour		Poisoning (100%)	
Read (1997) South Africa [141]	100 (25%/13–25)	Hospital	Clinician diagnosis, BDI, Hamilton, and the Montgomery-Asberg	Nonfatal suicidal behaviour		Poisoning (83%)	
Reza (2013) Bangladesh** [16]	113 (39%/All ages)	Community (self-report)	SCID	Nonfatal suicidal behaviour			
Rezaie (2011) Iran [142]	200 (34%/All ages*)	Hospital	Clinician diagnosis	Nonfatal suicidal behaviour		Poisoning (69%)	
Risal (2011) Nepal [143]	73 (30%/All ages)	Hospital	Clinician diagnosis	Nonfatal suicidal behaviour		Poisoning (93%)	
Santos (2009) Brazil [144]	96 (38%/All ages*)	Hospital	CIDI	Nonfatal suicidal behaviour	High	Drug overdose (40%)	
Sathish (2016) India [145]	50 (52%/All ages)	Hospital	MINI-plus	Nonfatal suicidal behaviour	High	Poisoning (60%)	
Seghatoleslam (2013) Iran [26]	292 (18%/6–15)	Hospital	Clinician diagnosis	Nonfatal suicidal behaviour		Drug overdose (89%)	
Shakeri (2015) Iran [146]	400 (0%/All ages)	Hospital	Clinician diagnosis	Nonfatal suicidal behaviour		Burning (44%)	
Sharma (1998) India [147]	75 (47%/All ages*)	Hospital	Clinician diagnosis	Nonfatal suicidal behaviour		Pesticide poisoning (75%)	
Sheikholeslami (2008) Iran [148]	575 (30%/All ages*)	Hospital	SCID-I and II	Nonfatal suicidal behaviour		Poisoning (90%)	
Simsek (2013) Turkey [149]	693 (22%/All ages)	Hospital	Clinician diagnosis	Nonfatal suicidal behaviour		Poisoning (90%)	
Srivastava (2004) India [150]	137 (47%/All ages*)	Hospital	Clinician diagnosis	Nonfatal suicidal behaviour		Poisoning	
Sun (2017) China [151]	791 (37%/15–54)	Hospital	SCID-I	Nonfatal suicidal behaviour	High	Pesticide poisoning (37%)	[152]
Thalagala (2003) Sri Lanka [153]	396 (54%/All ages*)	Hospital	Clinician diagnosis	Nonfatal suicidal behaviour			
Thanh (2005) Vietnam [154]	515 (37%/All ages)	Hospital	Clinician diagnosis	Nonfatal suicidal behaviour		Drug overdose (urban) and pesticide poisoning (rural)	
Tong (B) (2018) China [15]	120 (34%/≥18)	Hospital	SCID	Nonfatal suicidal behaviour	High	Pesticide poisoning (70%)	[54]
Toudehskchuie (2016) Iran [27]	240 (41%/All ages)	Hospital	SCID and alcohol dependency scale	Nonfatal suicidal behaviour		Drug overdose (73%)	
Trabelsi (2015) Tunisia [155]	33 (58%/All ages)	Hospital	Clinician diagnosis	Nonfatal suicidal behaviour		Drug overdose (58%)	
Tuan (2009) Vietnam [30]	309 (28%/All ages)	Hospital	CIDI	Nonfatal suicidal behaviour		Poisoning (100%)	
Unni (1995) India [156]	100 (%/≥10)	Hospital	Clinician diagnosis	Nonfatal suicidal behaviour			
van der Hoek (2005) Sri Lanka [32]	200 (61%/≥16)	Hospital	CIDI-SF	Nonfatal suicidal behaviour		Pesticide poisoning	
Vásquez-Rojas (2013) Colombia [157]	213 (26%/Children)	Hospital	Clinician diagnosis	Nonfatal suicidal behaviour		Poisoning	
Vishnuvardhan (2012) India [158]	100 (48%/≥18)	Hospital	SCID	Nonfatal suicidal behaviour		Pesticide poisoning (57%)	
Wang (2012) China [159]	59 (%/≥18)	Community (self-report)	SCID	Nonfatal suicidal behaviour	High		
Wei (2013) China [160]	239 (22%/≥15)	Hospital	SCID	Nonfatal suicidal behaviour		Poisoning (93%)	[161]
Wilson (1994) South Africa [24]	25 (76%/All ages)	Hospital	Clinician diagnosis	Nonfatal suicidal behaviour		Battery acid (100%)	
Xiao (2011) China [162]	617 (26%/All ages*)	Hospital	SCID	Nonfatal suicidal behaviour	High	Pesticide poisoning (76%)	[163–167]
Zarghami (2002) Iran** [17]	318 (17%/All ages)	Hospital	SCID	Nonfatal suicidal behaviour		Burning (100%)	

*Assumed all ages included.

**Study reports on both fatal and nonfatal suicidal behaviour as a combined outcome. The majority outcome (fatal/nonfatal) was used to class the study as either being a study of fatal or nonfatal suicidal behaviour.

**Abbreviations:** AUDIT, Alcohol use disorders identification test; BDI, Becks depression inventory; CIDI-SF, Composite international diagnostic interview–short form; CIS-R, Clinical interview schedule-revised; GAD-7, Generalised anxiety disorder assessment; MINI, Mini-international neuropsychiatric interview; PHQ-9, Patient health questionnaire; SADS, Schedule for affective disorders and schizophrenia; SCID, structured clinical interview for DSM

**Table 2:** Primary analysis of fatal suicidal behaviour studies by ICD-10 codes.

ICD-10 Diagnosis	Unrestricted			High-quality studies			
	No. estimates (No. of studies)	Pooled estimate (95% CI)	I^2^	No. estimates (No. of studies)	Pooled estimate (95% CI)	I^2^	Predictive interval
Any psychiatric disorder	19 (19)	57% (45%–69%)	98.6%	6 (6)	58% (46%–71%)	96.1%	13%-100%
Mental disorders due to known physiological conditions	3 (3)	−	−	1 (1)	−	−	
Mental and behavioural disorders due to psychoactive substance use	15 (11)	12% (8%–17%)	96.9%	4 (4)	−	−	
Schizophrenia, schizotypal, delusional, and other nonmood psychotic disorders	18 (15)	7% (5%–9%)	88.3%	5 (4)	8% (4%–12%)	85.8%	0%–23%
Mood [affective] disorders	22 (15)	24% (19%–29%)	99.0%	5 (4)	25% (8%–42%)	99.1%	0%–91%
Anxiety, dissociative, stress-related, somatoform, and other nonpsychotic mental disorders	12 (8)	10% (4%–15%)	96.5%	3 (2)	−	−	
Behavioural syndromes associated with physiological disturbances and physical factors	0 (0)	−	−	0 (0)	−	−	
Disorders of adult personality and behaviour	3 (3)	−	−	0 (0)	−	−	
Intellectual disabilities	3 (3)	−	−	0 (0)	−	−	
Pervasive and specific developmental disorders	0 (0)	−	−	0 (0)	−	−	
Behavioural and emotional disorders with onset usually occurring in childhood and adolescence	0 (0)	−	−	0 (0)	−	−	
Unspecified mental disorder	6 (6)	−	−	2 (2)	−	−	

**Abbreviation:** ICD, International Classification of Diseases

**Table 3:** Primary analysis of nonfatal suicidal behaviour studies by ICD-10 codes.

ICD-10 Diagnosis	Unrestricted			High-quality studies			
	No. estimates (No. of studies)	Pooled estimate (95% CI)	I^2^	No. estimates (No. of studies)	Pooled estimate (95% CI)	I^2^	Predictive interval
Any psychiatric disorder	59 (59)	55% (43%–67%)	99.9%	11 (11)	45% (30%–61%)	98.9%	0%–100%
Mental disorders due to known physiological conditions	9 (8)	2% (1%–4%)	83.3%	1 (1)	−	−	
Mental and behavioural disorders due to psychoactive substance use	60 (56)	8% (7%–9%)	96.0%	11 (9)	11% (7%–16%)	95.8%	0%–27%
Schizophrenia, schizotypal, delusional, and other nonmood psychotic disorders	66 (55)	5% (4%–6%)	95.2%	10 (8)	7% (3%–11%)	95.8%	0%–22%
Mood [affective] disorders	123 (79)	20% (18%–22%)	98.5%	17 (10)	21% (12%–29%)	98.4%	0%–54%
Anxiety, dissociative, stress-related, somatoform, and other nonpsychotic mental disorders	111 (55)	12% (10%–15%)	99.1%	14 (8)	19% (1%–36%)	99.8%	0%–88%
Behavioural syndromes associated with physiological disturbances and physical factors	5 (3)	3% (0%–5%)	44.5%	0 (0)	−	−	
Disorders of adult personality and behaviour	42 (33)	9% (6%–11%)	97.5%	4 (2)	−	−	
Intellectual disabilities	4 (4)	−	−	1 (1)	−	−	
Pervasive and specific developmental disorders	2 (2)	−	−	0 (0)	−	−	
Behavioural and emotional disorders with onset usually occurring in childhood and adolescence	7 (5)	3% (1%–4%)	80.9%	0 (0)	−	−	
Unspecified mental disorder	8 (8)	−	−	2 (2)	−	−	

**Abbreviation:** ICD, International Classification of Diseases

### Fatal suicidal behaviour

#### Primary analysis

**Fig 2:**
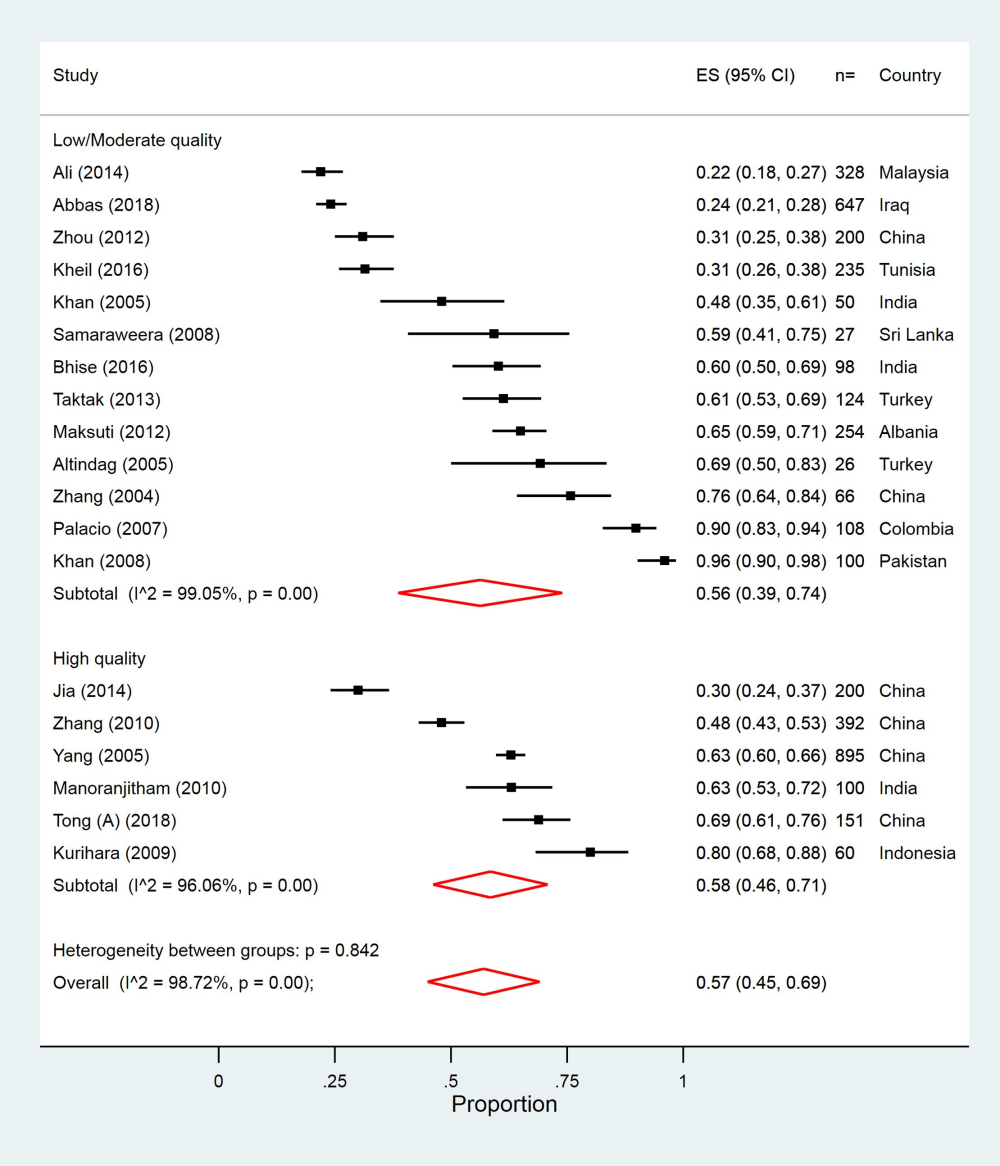
Overall prevalence of psychiatric disorder in high versus low/moderate quality studies of fatal suicidal behaviour. ES, Effect size.

Nineteen studies (out of 23) reported on an overall prevalence of psychiatric disorder for suicide deaths. Only 6 studies (from China, Indonesia, and India) were rated highly in our quality rating, and these studies included 1,798 individuals. There was a high degree of heterogeneity between the studies that were included in the meta-analysis (I^2^ = 96.1%), with estimates ranging from 30% [45] to 80% [48] (Table 2 and Fig 2).The pooled estimate of any psychiatric disorder in fatal suicidal behaviour, from these studies, was 58% (95% CI 46%–71%; Table 2 and Fig 2). There was at least one study for 8 of the 11 ICD-10 diagnosis categories, but only 2 categories had a sufficient number of high-quality studies to be included in the meta-analysis (Table 2). The unrestricted pooled estimates by each ICD-10 diagnosis category are summarised in Table 2 and shown in Fig A–D and L in S2 Appendix. Based on the evidence from high-quality studies, the most prevalent psychiatric disorder associated with fatal suicidal behaviour was mood disorder (25%).

#### Sensitivity analysis

We planned 3 subgroup analyses by region, proportion of males, and ICD-10 subgroups. We also conducted an additional analysis by the ages of individuals included in the studies and the assessment type. Given the limited number of studies, many of the subgroup analyses were unable to be conducted. These results are presented in detail in the supplementary material (Table A–E in S2 Appendix). There was little difference observed in the prevalence estimates, with the exception of the prevalence of anxiety-related disorders in which studies that were based on a clinician’s diagnosis (3%, 95% CI 1%–4%) reported lower rates than interview-based studies (14%, 95% CI 4%–24%). Only a limited amount of heterogeneity was explained by the sensitivity analysis.

### Nonfatal suicidal behaviour

#### Primary analysis

**Fig 3:**
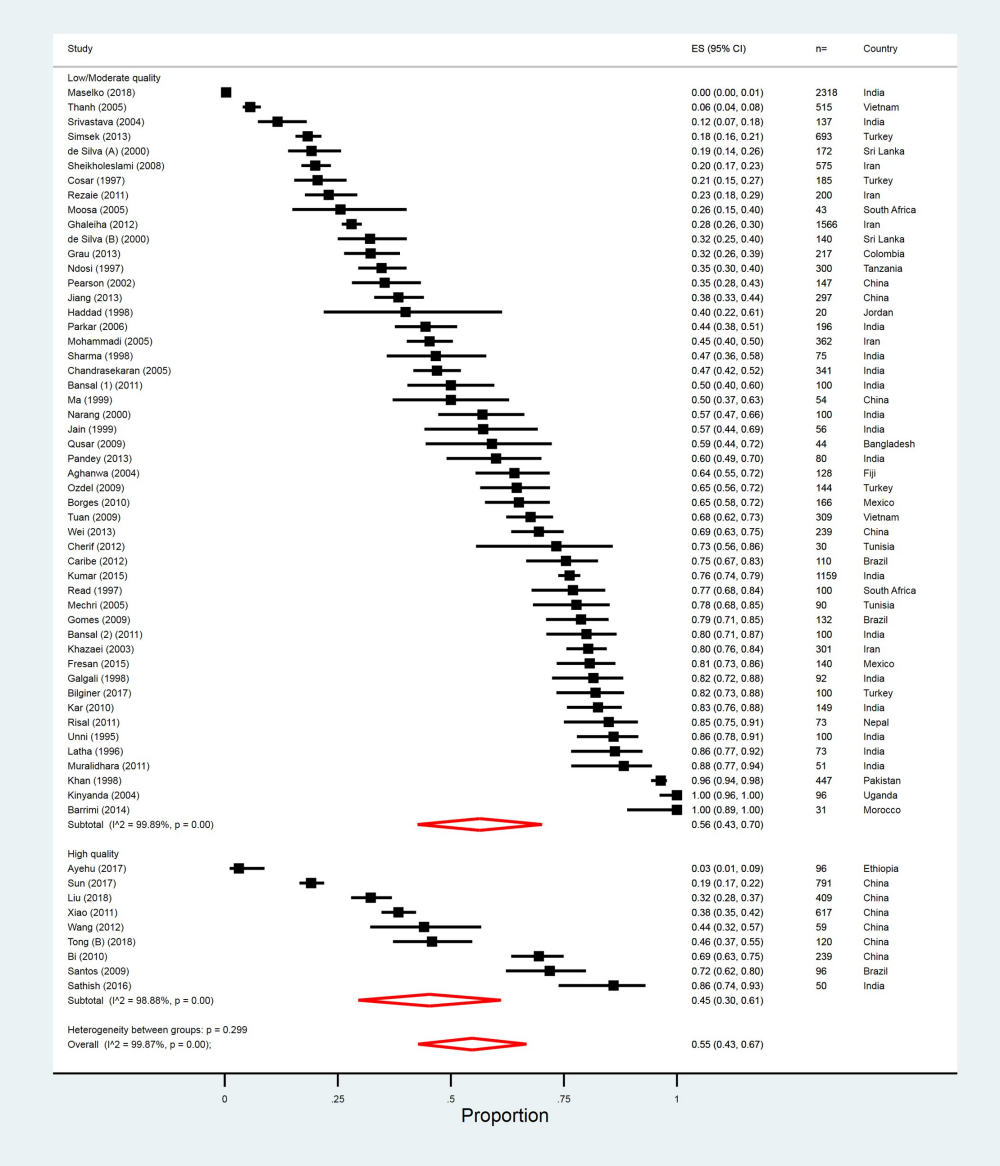
Overall prevalence of psychiatric disorder in high versus low/moderate quality studies of nonfatal suicidal behaviour. ES, Effect size.

A total of 59 studies (out of 89) reported on the total prevalence of mental disorders in nonfatal suicidal behaviour. There were only 9 high-quality studies (from Brazil, China, India, and Ethiopia), which gave an overall prevalence of psychiatric disorders of 45% (95% CI 30%–61%), with evidence of substantial heterogeneity between studies (I^2^ = 98.9%; Table 3 and Fig 3). These studies included 2,477 individuals. Estimates ranged between 3% [85] to 86% [145]. All 11 ICD-10 diagnosis categories had at least one study estimate, but there were only sufficient high-quality rated study estimates to generate pooled estimates for 4 of the categories (Table 3 and Fig E–L in S2 Appendix). The most prevalent disorder was mood disorder (21%), followed by anxiety and related disorders (17%).

#### Sensitivity analysis

Details of the sensitivity analysis are presented in the supplementary results (Table F–J in S2 Appendix). A limited amount of heterogeneity was explained by these analyses. We noted that the overall prevalence of psychiatric morbidity was lower in the East Asia and Pacific region (44%, 95% CI 30%–57%) than the other regions (range 50%–68%). Subgrouping by region reduced the amount of observed heterogeneity between studies for a limited number of ICD-10 categories. We also observed that studies with a higher proportion of male participants (i.e., ≥50% males) tended to have a higher prevalence of overall psychiatric morbidity (74%, 95% CI 63%–84%) compared with studies with less than 50% male participants (47%, 95% CI 36%–59%). This pattern persisted for all but one ICD-10 category. There were, however, only 8 studies that presented sex stratified prevalence of psychiatric morbidity from India, China, and Iran [91,102,117,121,135,148,151,160]. Two studies reported a higher prevalence of psychiatric morbidity in males than females [121,135], one reported a higher prevalence in females [117], and the remaining 5 studies reported no differences [91,102,148,151,160]. Studies that used a clinician’s diagnosis of psychiatric morbidity reported a higher prevalence of anxiety related disorders (15%, 95% CI 11%–19%) than those that used a validated diagnostic interview (8%, 95% CI 7%–10%). We found that studies based on self-reported cases of nonfatal suicidal behaviour reported lower levels of mood disorders (12%, 95% CI 9%–16%) compared with other studies (21%, 95% CI 18%–23%). This was also observed for anxiety related disorders (self-report: 8%, 95% CI 5%–11%); other: 13%, 95% CI 10%–15%), but the confidence intervals of the pooled estimates overlapped. We were unable to conduct a subgroup analysis by type of case ascertainment because of too few self-report studies that reported any psychiatric morbidity.

## Discussion

To our knowledge, this is the first systematic review and meta-analysis focussed on psychiatric morbidity in suicidal behaviour in LMIC. A total of 112 studies in 26 LMIC have allowed us to estimate the prevalence of psychiatric morbidity in individuals who have engaged in suicidal behaviour. There were only 17 studies that were considered high-quality, with substantial heterogeneity observed between study estimates for psychiatric disorders. Prevalence estimates for psychiatric disorders in individuals who died by suicide ranged between 30% and 80% and between 3% and 86% in those who engaged in nonfatal suicidal behaviour. The pooled estimate of psychiatric disorders was 58% and 45% in fatal and nonfatal suicidal behaviour, respectively, though these estimates need to be interpreted with caution given the high degree of heterogeneity. The most prevalent disorder in both fatal and nonfatal suicidal behaviour was mood disorder, which was identified in roughly 1 in 4 cases. We also found that studies with a higher proportion of males reported higher levels of psychiatric disorders than studies with fewer males.

This evidence synthesis suggests that psychiatric disorders may be less common in individuals who engage in suicidal behaviour in LMIC. This result needs to be interpreted in light of the high degree of heterogeneity between study estimates and the wide-ranging estimates identified. The estimated prevalence in this review is markedly lower than the estimated prevalence in HIC (80%–92% [3,4,6]), but consistent with the prevalence of psychiatric disorders in ‘non-western’ (LMIC but also including Japan and Hong Kong) countries included in a previous review (fatal: 70%, 95% CI 57%–80% [4]; nonfatal: 59%, 95% CI 57%–61% [6]). The possible lower prevalence of psychiatric disorders in LMIC could reflect the suicide methods (e.g., pesticide ingestion) used in impulsive acts of self-harm with low suicidal intent that may be less strongly associated with mental illness [168]. It has also been suggested that suicidal behaviour may function as a means of communication in LMIC by disempowered members of the community (e.g., women and young people). When it is used in this way, it may again be less likely to be associated with psychiatric morbidity [169]. In addition, there may be culturally specific expressions of psychiatric symptoms that are not captured by diagnostic criteria such as ICD and DSM [170].

The most prevalent psychiatric disorder in this review, for both fatal and nonfatal suicidal behaviour, was a mood (affective) disorder (25% and 21%, respectively). This is much lower than the estimates in previous reviews of primarily HIC for suicide and nonfatal suicidal behaviour (43%–59%) [3–6]. The prevalence of other psychiatric disorder diagnoses for nonfatal suicidal behaviour was considerably lower in this study compared with a previous meta-analysis [6] (disorder [this study versus previous review]: anxiety disorders [19% versus 35%], substance misuse [11% versus 34%], and mood disorders [21% versus 59%]). This difference is likely to be explained by the fact that 70% to 80% of studies included in the previous reviews were from HIC. Only psychotic disorders had a similar prevalence (6%–9%) [4–6].

In this review, we also observed a suggested lower prevalence of psychiatric disorders in studies that included fewer males than females. There were very few studies that reported on sex specific prevalence rates, and whilst some supported our findings of a higher rate in males [121,135], others did not [91,102,117,121,135,148,151,160].

Of the 112 studies identified for this review, just under a fifth were considered high-quality studies that collected consecutive cases of suicidal behaviour (regardless of method) and that used a standardised interview schedule to obtain diagnosis. We considered studies that used a standardised interview schedule to be of higher quality because they are more likely to capture undiagnosed mental disorders. Under-recognition may be more likely in LMIC because of the limited number of mental health professionals who might formally diagnose psychiatric illness. Studies using clinician diagnoses did not generate very different results, apart for anxiety disorder (lower prevalence than diagnostic interviews in fatal behaviour, higher prevalence in nonfatal behaviour). Studies did not always present sex-specific rates of psychiatric disorder, nor did they present data in such a way to allow us to investigate how many patients had more than one diagnosis. We were also unable to distinguish between self-harm with and without intent in the studies included in the review, nor were we able to categorise studies according to whether attempts were first time or repeat episodes. For some studies, certain contextual factors may have impacted on the generalisability of the study findings to the wider suicidal population. For example, in countries where suicidal behaviour is still (or recently) considered a criminal act [171], the ascertainment of cases may be subject to a selection bias, with more medically serious attempts being included [16,40,47,50,86,87,93,95,105,106,108,110,111,113,114,118–120,129,132,135,136,140,145,147,150,156,158]. There is a clear need for more high-quality studies investigating the association between psychiatric morbidity and suicidal behaviour.

According to our findings, around half of individuals who engaged in suicidal behaviour had a psychiatric disorder in LMIC compared with most in HIC [3,6]. There was, however, a high degree of heterogeneity between studies and wide-ranging prevalence estimates. This heterogeneity could be because of differences in methodology. The studies themselves varied in ascertainment of outcomes, explanatory variables, and recruitment methods. Other possible sources of heterogeneity between studies may have arisen from variations in the way diagnostic criteria were interpreted and applied or the qualifications and training of interviewers (e.g., lay versus mental health professionals) [170]. Future studies should aim to recruit consecutive cases of suicidal behaviour regardless of method used and assess psychiatric disorders using standardised instruments that have cross-cultural validity. However, the heterogeneity could also reflect real differences in the aetiology of suicidal behaviour and prevalence of psychiatric disorder between countries. We explored the possibility of this by grouping studies by region, but the heterogeneity within regions remained high. It is clear that not all LMIC are the same. This raises a more fundamental question about whether the current LMIC/HIC dichotomy is meaningful. It might be more helpful to group countries according to contextual differences or by other indices (e.g., sociodemographic index or human development index).

In HIC, policy, clinical, and prevention interventions highlight the central role of psychiatric disorders. The possible lower prevalence of psychiatric disorders in suicidal behaviour in LMIC suggests that whilst the treatment of psychiatric disorders is important, it may need to be a part of a wider suite of prevention activities. In addition, it is important to note the interaction between local sociocultural factors (e.g., socially acceptable behaviours) and wider societal changes related to factors such as globalisation [172]. Prevention efforts in LMIC may need to consider addressing a number of modifiable factors along the causal pathway. Community-level factors, such as access to lethal means of suicide (e.g., pesticides), should be targets for suicide prevention. National bans of pesticides has been shown to be effective in reducing the number of suicide deaths [173]. Social and economic stressors, such as poverty [174,175], unemployment, and domestic violence [176], could be important targets for intervention. Measures to achieve poverty reduction include improved welfare support, opportunities for stable employment, and debt assistance [177]. Family conflicts are a key risk factor for suicide in Asian women [178,179]. Tackling domestic violence requires changes to the perceived status of women in communities, gender norms, and the negotiation of power in interpersonal relationships. These are ambitious aims, but a promising social change intervention has been trialled in Uganda [180]. The key elements included improved communication, supportive gender roles, joint decision-making, and nonviolent ways for dealing with relationship conflicts.

These community and nonmedical approaches may need to be part of the prevention activities in LMIC, alongside approaches that seek to modify the psychiatric risk factors. We found that half of suicidal behaviour was associated with mental disorder. How should mental health preventive efforts be focussed in LMIC? There is a substantial treatment gap in LMIC (with over 75% of psychiatric disorders going untreated [181]), and it is estimated that there is a workforce deficit of 11,000 psychiatrists in these settings [182]. Increasing the specialist mental health workforce in these contexts could be one priority, but the resources needed to do this might be limited in LMIC. One approach, which has proved effective in the treatment of depression and alcohol use disorders in India, is the provision of treatment by nonspecialist health workers (i.e., lay counsellors) [183,184]. Collaborative task sharing in these settings is a promising way forward in reducing the treatment gap, and, subsequently, in reducing the number of people who engage in suicidal behaviour.

To the best of our knowledge, this is the first systematic review and meta-analysis which has aimed to comprehensively synthesise existing evidence from LMIC in order to estimate the prevalence of psychiatric disorders in individuals who engaged in suicidal behaviour. A particular strength of this review is that no language restrictions were applied (this resulted in the inclusion of 18 papers that would otherwise have been excluded). In comparison to previous reviews, we consider both fatal and nonfatal suicidal behaviour within the same review. There were, however, limitations that need to be considered when interpreting our findings. There were few high-quality studies and substantial heterogeneity in our findings. We attempted to explore the sources of heterogeneity using our prespecified subgroup analysis but in almost all cases the heterogeneity remained high. We also relied on searches from journals indexed in 3 large databases but given that these tend to index primarily English language journals we may have missed publications. We attempted to overcome this limitation by conducting hand searches of key systematic reviews [3–6] and all included studies.

In conclusion, prevalence estimates for psychiatric disorders in individuals who died by suicide ranged between 30% and 80% and between 3% and 86% in those who engaged in nonfatal suicidal behaviour. Less than a fifth of identified studies were rated as high-quality, and substantial heterogeneity was observed between study estimates. Psychiatric morbidity may be less in suicidal behaviour in LMIC. There is, however, an urgent need for more methodologically robust studies from these settings to help these resource-poor countries to appropriately balance preventive approaches.

## Supporting information

S1 PRISMA Checklist PRISMA, Preferred Reporting Items for Systematic Reviews and Meta-Analyses. (DOC)

S1 Protocol PROSPERO registration of review protocol. PROSPERO, International prospective register of systematic reviews
(PDF)

S1 Data Extracted data used in review. (XLS)

S1 Appendix Supplementary methods. (DOCX)

S2 Appendix Supplementary results. (DOCX)

## Abbreviations

AUDIT: Alcohol use disorders identification test
BDI: Becks depression inventory
CIDI-SF: Composite international diagnostic interview–short form
CIS-R: Clinical interview schedule-revised
ES: Effect size
GAD-7: Generalised anxiety disorder assessment
HIC: high-income countries
ICD: International Classification of Diseases
LMIC: low- and middle-income countries
MINI: Mini-international neuropsychiatric interview
NOS: Newcastle-Ottawa scale
NSSI: nonsuicidal self-injury
PHQ-9: Patient health questionnaire
SADS: Schedule for affective disorders and schizophrenia
SCID: structured clinical interview for DSM
