# Supplementary material for: Psychiatric morbidity and suicidal behaviour in low- and middle-income countries: A systematic review and meta-analysis
Source: PLoS Med. 2019 Oct 9;16(10):e1002905. doi: 10.1371/journal.pmed.1002905 (PMC6785653; doi:10.1371/journal.pmed.1002905)
Supplement: S1 Protocol — PROSPERO, International prospective register of systematic reviews (PDF) [file pmed.1002905.s002.pdf]

NHS
National Institute for Health Research

Psychiatric morbidity and suicide in low and middle-income countries

Duleeka Knipe, Nav Kapur, Stephanie Hannam-Swain, Katherine Brown, A. Jess Williams

Citation

Duleeka Knipe, Nav Kapur, Stephanie Hannam-Swain, Katherine Brown, A. Jess Williams. Psychiatric morbidity and suicide in low and middle-income countries. PROSPERO 2018 CRD42018087851 Available from: http://www.crd.york.ac.uk/PROSPERO/display_record.php?ID=CRD42018087851

Review question

What is the prevalence of psychiatric morbidity in individuals who self-harm (fatal and non-fatal) in low and middle-income countries?

Searches

We will search the following electronic bibliographic databases: MEDLINE, EMBASE, and PsycINFO for reports of the prevalence of psychiatric morbidity in individuals who self-harmed (both fatal and non-fatal) regardless of suicidal intent. We will search the reference lists of eligible papers and conduct citation searches of key papers to identify additional reports.

Papers in English or with English language abstracts will be eligible to be included. Human studies published between January 1990 and the date the searches are run will be sought.

Types of study to be included

Case series, case-control studies, cohort studies and randomised control trials (results extracted for control arm).

Condition or domain being studied

Self harm (fatal and non-fatal) regardless of suicidal intent

Participants/population

INCLUSION: The populations included in this review will be restricted to studies in low and middle income countries (LMIC) (http://data.worldbank.org/about/country-classifications) in humans. Only individuals who have either presented with or self-identify as having engaged in self-harming behaviour (fatal or non-fatal) will be included. No age or gender restrictions will apply. Priority will be given to general population studies, but we may include demographic and other subgroups based on representativeness.

EXCLUSIONS: Those who report only suicide ideation will not be included in this review. Subjective impressions of psychiatric morbidity (i.e. without a clinical diagnosis or assessed via a validated rating scale) will be excluded. Studies which only report on lifetime non-fatal self-harm and/or lifetime psychiatric morbidity will be excluded.

Intervention(s), exposure(s)

Not applicable. The review is interested in obtaining an estimate of prevalence of psychiatric morbidity in individuals who self-harm (fatal and non-fatal)

Comparator(s)/control

Not applicable. The review is interested in obtaining an estimate of prevalence of psychiatric morbidity in individuals who self-harm (fatal and non-fatal)

Context

Studies in low and middle income countries (LMIC) only (http://data.worldbank.org/about/country-classifications)

Main outcome(s)

Assessment of the prevalence of psychiatric morbidity (conditions that fall under the ICD-10 F01-99 codes)

in individuals who self-harm. Psychiatric morbidity is defined as a clinical diagnosis or reach a threshold for caseness on a validated interview or scale.

Additional outcome(s)

None

Data extraction (selection and coding)

Titles and/or abstracts of studies retrieved using the search strategy and those from additional sources will be screened to identify studies that potentially meet the inclusion criteria outlined above. The full text of these potentially eligible studies will be retrieved and independently assessed for eligibility by two review team members. Any disagreement between them over the eligibility of particular studies will be resolved through discussion with a third reviewer.

A structured, pre-piloted form will be used to extract data from the included studies for assessment of study quality and evidence synthesis. Extracted information will include: study design/setting; study population, and participant demographics/characteristics (e.g age, sex); type of self-harm; type of outcome measure and definition (i.e. cut-off); reported prevalence (n %); and information for assessment of the risk of bias (see below). Two review authors will extract data independently, discrepancies will be identified and resolved through discussion (with a third author where necessary).

Risk of bias (quality) assessment

The overall quality of each study will be evaluated and rated according to the Newcastle-Ottawa scale (NOS) and a modified NOS version for cross-sectional studies.

Strategy for data synthesis

If the studies identified in this review are suitable for inclusion (i.e. are not too heterogeneous) in a meta-analysis, a pooled estimate of the prevalence of any psychiatric disorder (and by type) in individuals who self-harm will be presented. If data are not homogeneous for a quantitative synthesis a narrative (descriptive) synthesis will be presented.

Analysis of subgroups or subsets

If a meta-analysis is conducted we will explore any heterogeneity between studies by pre-specified groupings, which will include: study design, region, sex, outcome (fatal vs. non-fatal), type of psychiatric condition, outcome measure, and NOS risk of bias.

Contact details for further information

Duleeka Knipe
dee.knipe@bristol.ac.uk

Organisational affiliation of the review

University of Bristol

Review team members and their organisational affiliations

Dr Duleeka Knipe. University of Bristol
Professor Nav Kapur. University of Manchester
Ms Stephanie Hannam-Swain. Sheffield Hallam University
Ms Katherine Brown. University of Nottingham
Ms A. Jess Williams. Nottingham Trent University

Type and method of review

Epidemiologic, Systematic review

Anticipated or actual start date

01 March 2018

Anticipated completion date

31 August 2018

Funding sources/sponsors

No funding

Conflicts of interest

Language
English

Country
England

Stage of review
Review Ongoing

Subject index terms status
Subject indexing assigned by CRD

Subject index terms
Humans; Income; Morbidity; Suicide

Date of registration in PROSPERO
21 February 2018

Date of publication of this version
29 August 2018

Details of any existing review of the same topic by the same authors

Stage of review at time of this submission

Stage	Started	Completed
Preliminary searches	Yes	No
Piloting of the study selection process	No	No
Formal screening of search results against eligibility criteria	No	No
Data extraction	No	No
Risk of bias (quality) assessment	No	No
Data analysis	No	No

Versions
21 February 2018
29 August 2018

PROSPERO
This information has been provided by the named contact for this review. CRD has accepted this information in good faith and registered the review in PROSPERO. The registrant confirms that the information supplied for this submission is accurate and complete. CRD bears no responsibility or liability for the content of this registration record, any associated files or external websites.
