# Supplementary material for: Psychiatric morbidity and suicidal behaviour in low- and middle-income countries: A systematic review and meta-analysis
Source: PLoS Med. 2019 Oct 9;16(10):e1002905. doi: 10.1371/journal.pmed.1002905 (PMC6785653; doi:10.1371/journal.pmed.1002905)
Supplement: S2 Appendix — (DOCX) [file pmed.1002905.s005.docx]

**Supplementary results**

**Fatal suicidal behaviour sensitivity analysis**

*Region-specific sub-group analysis*: we were only able to generate a pooled estimate for the total prevalence of psychiatric disorders for East Asia and the Pacific, and South Asia region, as there were too few studies (<5 studies) from the other regions. We were also only able to generate pooled estimates for the regions of East Asia and Pacific, Middle East and North Africa, and South Asia for 4 of the ICD-10 categories (see supplementary table A below). Only a limited amount of heterogeneity was explained by this sensitivity analysis.

**Supplementary table A – Prevalence of** **psychiatric disorders of fatal suicidal behaviour by region**

|  | East Asia & Pacific | | Middle East & North Africa | | South Asia | |
| --- | --- | --- | --- | --- | --- | --- |
|  | Pooled estimate (95% CI) | I^2^ | Pooled estimate (95% CI) | I^2^ | Pooled estimate (95% CI) | I^2^ |
| Any psychiatric disorder | 52% (37-67%) | 98.1 | - | - | 66% (44-88%) | 96.4 |
| Mental and behavioural disorders due to psychoactive substance use | - | - | - | - | 12% (3-22%) | 98.9 |
| Schizophrenia, schizotypal, delusional, and other non-mood psychotic disorders | 8% (6-11%) | 67.8 | - | - | 6% (2-9%) | 79.8 |
| Mood [affective] disorders | 26% (12-41%) | 99.2 | 8 (3-13%) | 96.9 | 30% (14-45%) | 99.1 |
| Anxiety, dissociative, stress-related, somatoform and other nonpsychotic mental disorders |  |  |  |  | 5% (1-9%) | 76.9 |

*Proportion of males sub-group analysis:* There were too few overall prevalence estimates of psychiatric morbidity in studies with less than 50% male deaths for our subgroup analysis. This was also the case for all of the ICD-10 categories. We present the restricted analysis for the studies which had more than 50% male deaths (Supplementary table B).

**Supplementary table B – Prevalence of** **psychiatric disorders of fatal suicidal behaviour in studies with ≥50% male suicide deaths**

|  | ≥50% males | |
| --- | --- | --- |
|  | Pooled estimate (95% CI) | I^2^ |
| Any psychiatric disorder | 59% (45-72%) | 98.9 |
| Mental and behavioural disorders due to psychoactive substance use | 14% (9-20%) | 97.4 |
| Schizophrenia, schizotypal, delusional, and other non-mood psychotic disorders | 8% (5-10%) | 88.3 |
| Mood [affective] disorders | 26% (21-32%) | 99.0 |
| Anxiety, dissociative, stress-related, somatoform and other nonpsychotic mental disorders | 5% (3-8%) | 68.6 |

*F-code sub-group analysis*: In the final prespecified sub analysis, we grouped the ICD-10 diagnoses into further sub-categories. We only did this for ICD-10 codes which had enough studies to allow for further categorisation. There was still a substantial degree of heterogeneity that persisted in these subgroup analyses (Supplementary table C).

**Supplementary table C– Prevalence of** **psychiatric disorders of fatal suicidal behaviour by F-code subgroupings**

|  |  | Pooled estimate (95% CI) | I^2^ |
| --- | --- | --- | --- |
| Mental and behavioural disorders due to psychoactive substance use | Alcohol dependence/use | 13% (4-22%) | 97.5 |
|  | Substance dependence* | 12% (7-17%) | 96.2 |
| Schizophrenia, schizotypal, delusional, and other non-mood psychotic disorders | Schizophrenia | 8% (5-10%) | 82.3 |
|  | Related disorders | 5% (2-8%) | 86.5 |
| Mood [affective] disorders | Depression | 29% (20-37%) | 98.7 |
|  | Bipolar disorder | - | - |
|  | Mood disorder | - | - |
| Anxiety, dissociative, stress-related, somatoform and other nonpsychotic mental disorders | Anxiety | - | - |
|  | PTSD | - | - |
|  | Somatoform disorder | - | - |
|  | Adjustment disorders | 13% (3-22%) | 97.7 |
|  | OCD | - | - |

* Includes both any substance use and non-alcohol substance use

*Age-group specific sub-group analysis*: Whilst we aimed to group studies into three categories based on the ages of individuals included in the studies, we found that the review only yielded enough studies (i.e. more than 5 studies in each sub-group) to generate pooled estimates for studies in working age adults or included individuals of all ages. There were insufficient studies to generates pooled estimates by age sub-group for all ICD-10 codes. A substantial degree of heterogeneity persisted in this sensitivity analysis (Supplementary table D).

**Supplementary table D – Prevalence of** **psychiatric disorders of fatal suicidal behaviour in studies of working age adults (26-65 years) and all ages**

|  | Working age adults/All Ages | |
| --- | --- | --- |
|  | Pooled estimate (95% CI) | I^2^ |
| Any psychiatric disorder | 57% (45-69%) | 98.7 |
| Mental and behavioural disorders due to psychoactive substance use | 12% (8-17%) | 96.9 |
| Schizophrenia, schizotypal, delusional, and other non-mood psychotic disorders | 7% (5-9%) | 88.3 |
| Mood [affective] disorders | 24% (19-29%) | 99.0 |
| Anxiety, dissociative, stress-related, somatoform and other nonpsychotic mental disorders | 10 (4-15%) | 96.2 |

*Assessment type sub-group analysis*: There were too few studies that used a validated scale only to assess psychiatric morbidity, therefore our analysis was restricted to comparing a clinician diagnosis with a validated diagnostic interview. There were no noticeable differences between the two assessment types (overlapping confidence intervals) for the prevalence of any psychiatric disorder and mood disorders. Studies that used a validated interview reported a higher prevalence of anxiety and related disorders, then studies that were based on a clinician’s diagnosis. There was a significant reduction in the heterogeneity observed in studies based on a clinical diagnosis, whilst the heterogeneity persisted for the other disorders.

**Supplementary table E – Prevalence of** **psychiatric disorders of fatal suicidal behaviour in studies that used clinician diagnosis versus a validated diagnostic interview**

|  | Clinician Diagnosis | | Validated interview | |
| --- | --- | --- | --- | --- |
|  | Pooled estimate (95% CI) | I^2^ | Pooled estimate (95% CI) | I^2^ |
| Any psychiatric disorder | 57% (37-77%) | 99.2 | 57% (45-69%) | 96.5 |
| Mood [affective] disorders | 29% (23-36%) | 99.2 | 20% (11-29%) | 98.5 |
| Anxiety, dissociative, stress-related, somatoform and other nonpsychotic mental disorders | 3% (1-4%) | 18.6 | 14% (4-24%) | 97.9 |

**Non-fatal suicidal behaviour sensitivity analysis**

*Region-specific sub-group analysis*: For this sensitivity analysis we used a slightly different method for pooling estimates to the main analysis as the method used in the main analysis generated inadmissible pooled estimates as study estimates were close to the margin. For this analysis we used the Freeman-Tukey double arcsine transformation to compute pooled estimates.

We were unable to generate a pooled estimate for the total prevalence of mental disorders in Europe and Central Asia but were able to create region specific estimates for the other regions (Supplementary Table F). Compared to the other regions, the prevalence estimate for any mental disorder was lower in East Asia and the Pacific region (44%). We were able to generate region specific pooled estimates for 5 of the ICD-10 categories, with little evidence that the estimates differed by region. The exception to this, is the slight indication that Latin American and Caribbean, and Middle East and North African countries had a lower pooled estimate for mood disorders than the other regions. Whilst a limited amount of heterogeneity was explained by this sensitivity analysis for overall mental disorders, there was some indication that subgrouping by region reduced heterogeneity at least within certain regions for particular ICD-10 diagnoses.

**Supplementary table F – Prevalence of** **psychiatric disorders of non-fatal suicidal behaviour by region**

|  | East Asia & Pacific | | Europe & Central Asia | | Latin America & Caribbean | | Middle East & North Africa | | South Asia | | Sub-Saharan Africa | |
| --- | --- | --- | --- | --- | --- | --- | --- | --- | --- | --- | --- | --- |
|  | Pooled estimate (95% CI) | I^2^ | Pooled estimate (95% CI) | I^2^ | Pooled estimate (95% CI) | I^2^ | Pooled estimate (95% CI) | I^2^ | Pooled estimate (95% CI) | I^2^ | Pooled estimate (95% CI) | I^2^ |
| Any psychiatric disorder | 44% (30-57%) | 98.6 | - | - | 68% (51-83%) | 99.2 | 56% (38-72%) | 98.6 | 61% (38-82%) | 99.6 | 50% (12-88%) | 99.0 |
| Mental and behavioural disorders due to psychoactive substance use | 5% (2-10%) | 95.9 | - | - | 10% (5-16%) | 91.1 | 10% (2-23%) | 96.5 | 8% (6-10%) | 89.6 | 4% (2-6%) | 15.4 |
| Schizophrenia, schizotypal, delusional, and other non-mood psychotic disorders | 6% (3-9%) | 95.7 | 9% (2-20%) | 94.4 | 5% (2-10%) | 87.0 | 4% (2-8%) | 90.6 | 4% (3-5%) | 65.3 | - | - |
| Mood [affective] disorders | 20% (12-28%) | 98.7 | 24% (13-37%) | 95.1 | 12% (6-19%) | 96.3 | 14% (9-20%) | 97.2 | 20% (15-26%) | 97.5 | 24 (7-46%) | 98.2 |
| Anxiety, dissociative, stress-related, somatoform and other nonpsychotic mental disorders | 8% (1-22%) | 99.6 | 13% (7-19%) | 71.6 | 11% (5-19%) | 97.1 | 10% (5-16%) | 98.4 | 10% (6-15%) | 97.4 | 8% (2-16%) | 93.1 |
| Disorders of adult personality and behaviour | 4% (2-5%) | 0.0 | - | - | - | - | 8% (6-10%) | 28.1 | 7% (3-14%) | 96.9 | - | - |

*Proportion of males sub-group analysis:* Not all the studies from the review had data on the proportion of males included, and therefore not all studies are included in this sub-analysis. We found evidence that the studies with a greater proportion of male participants had a higher prevalence of psychiatric disorders (74%) associated with the act than in studies with less than 50% male participants (47%) (Supplementary table G). This lower prevalence was observed in most the ICD-10 categories where we were able to generate subgroup pooled estimates. There was a slight reduction in the heterogeneity observed in the subgroups, although substantial heterogeneity between studies persisted, except for the disorders of adult personality and behaviour.

**Supplementary table G – Prevalence of** **psychiatric disorders of non-fatal suicidal behaviour by proportion of males**

|  | <50% males | | ≥50% males | |
| --- | --- | --- | --- | --- |
|  | Pooled estimate (95% CI) | I^2^ | Pooled estimate (95% CI) | I^2^ |
| Any psychiatric disorder | 47% (36-59%) | 99.8 | 74% (63-84%) | 98.4 |
| Mental and behavioural disorders due to psychoactive substance use | 3% (1-5%) | 99.8 | - | - |
| Schizophrenia, schizotypal, delusional, and other non-mood psychotic disorders | 6% (5-7%) | 92.6 | 10% (8-13%) | 80.1 |
| Mood [affective] disorders | 4% (3-5%) | 86.1 | 4% (3-6%) | 73.8 |
| Anxiety, dissociative, stress-related, somatoform and other nonpsychotic mental disorders | 18% (16-20%) | 98.2 | 25% (20-29%) | 97.7 |
| Behavioural syndromes associated with physiological disturbances and physical factors | 10% (8-11%) | 97.7 | 13% (10-16%) | 97.1 |
| Disorders of adult personality and behaviour | 3% (0-5%) | 44.7 | - | - |

*F-code sub-group analysis*: We conducted a sub-group analysis of ICD-10 codes for 4 of the ICD10 categories. The subgroup analysis explained some of the heterogeneity in the anxiety and related disorders category (Supplementary table H). However, for most of the ICD10 categories the subgrouping made little difference to the observed heterogeneity.

**Supplementary table H – Prevalence of** **psychiatric disorders of non-fatal suicidal behaviour by F-code subgroupings**

|  |  | Pooled estimate (95% CI) | I^2^ |
| --- | --- | --- | --- |
| Mental and behavioural disorders due to psychoactive substance use | Alcohol dependence/use | 7% (5-8%) | 92.9 |
|  | Substance dependence | 9% (7-11%) | 95.2 |
| Schizophrenia, schizotypal, delusional, and other non-mood psychotic disorders | Schizophrenia | 4% (3-5%) | 80.2 |
|  | Related disorders | 5% (3-8%) | 97.8 |
| Mood [affective] disorders | Depression | 25% (21-28%) | 98.1 |
|  | Bipolar disorder | 5% (3-7%) | 96.5 |
|  | Mood disorder | 22% (16-28%) | 99.3 |
| Anxiety, dissociative, stress-related, somatoform and other nonpsychotic mental disorders | Anxiety | 10% (5-15%) | 99.6 |
|  | PTSD | 2% (1-4%) | 86.1 |
|  | Somatoform disorder | 1% (0-1%) | 14.5 |
|  | Adjustment disorders | 23% (17-28%) | 98.6 |
|  | OCD | 3% (2-5%) | 70.9 |

*Age-group specific sub-group analysis*: There were insufficient studies to generates pooled estimates by age sub-group for all ICD-10 codes, and only enough studies to generate pooled estimates for the working age adults/all age sub-group. A substantial degree of heterogeneity persisted in this sensitivity analysis for most ICD-10 codes, except for mental disorders due to known physiological conditions (Supplementary table I).

**Supplementary table I – Prevalence of** **psychiatric disorders of non-fatal suicidal behaviour by age group**

|  | Young people (≤25 years) | | Working age adults/All Ages | |
| --- | --- | --- | --- | --- |
|  | Pooled estimate (95% CI) | I^2^ | Pooled estimate (95% CI) | I^2^ |
| Any psychiatric disorder | - | - | 54% (41-66%) | 99.9 |
| Mental disorders due to known physiological conditions | - | - | 1% (0-2%) | 37.0 |
| Mental and behavioural disorders due to psychoactive substance use | - | - | 8% (7-10%) | 96.2 |
| Schizophrenia, schizotypal, delusional, and other non-mood psychotic disorders | - | - | 5% (4-6%) | 95.4 |
| Mood [affective] disorders | 21% (11-32%) | 97.6 | 20 (18-22%) | 98.6 |
| Anxiety, dissociative, stress-related, somatoform and other nonpsychotic mental disorders | 18% (10-26%) | 99.3 | 12% (9-14%) | 99.1 |
| Disorders of adult personality and behaviour | - | - | 9% (6-12%) | 97.7 |

*Assessment type sub-group analysis*: As with fatal suicidal behaviour, there were too few studies that used a validated scale only to assess psychiatric morbidity, therefore our analysis was restricted to comparing a clinician diagnosis with a validated diagnostic interview. There were no noticeable differences between the two assessment types for the prevalence of any psychiatric disorder and mood disorders. Studies that used a validated interview reported a lower prevalence of anxiety and related disorders, then studies that were based on a clinician’s diagnosis. Little of the heterogeneity was explained in this subgroup analysis.

**Supplementary table J – Prevalence of** **psychiatric disorders of non-fatal suicidal behaviour in studies that used clinician diagnosis versus a validated diagnostic interview**

|  | Clinician Diagnosis | | Validated interview | |
| --- | --- | --- | --- | --- |
|  | Pooled estimate (95% CI) | I^2^ | Pooled estimate (95% CI) | I^2^ |
| Any psychiatric disorder | 59% (46-72%) | 99.70 | 49% (37-61%) | 99.6 |
| Mood [affective] disorders | 20% (17-22%) | 98.50 | 20% (16-23%) | 97.9 |
| Anxiety, dissociative, stress-related, somatoform and other nonpsychotic mental disorders | 15% (11-19%) | 99.50 | 8% (7-10%) | 94.9 |

**Supplementary figures**

**Supplementary fig A – Prevalence of** **mental and behavioural disorders due to psychoactive substance use in high vs. low/moderate quality studies of fatal suicidal behaviour**

**
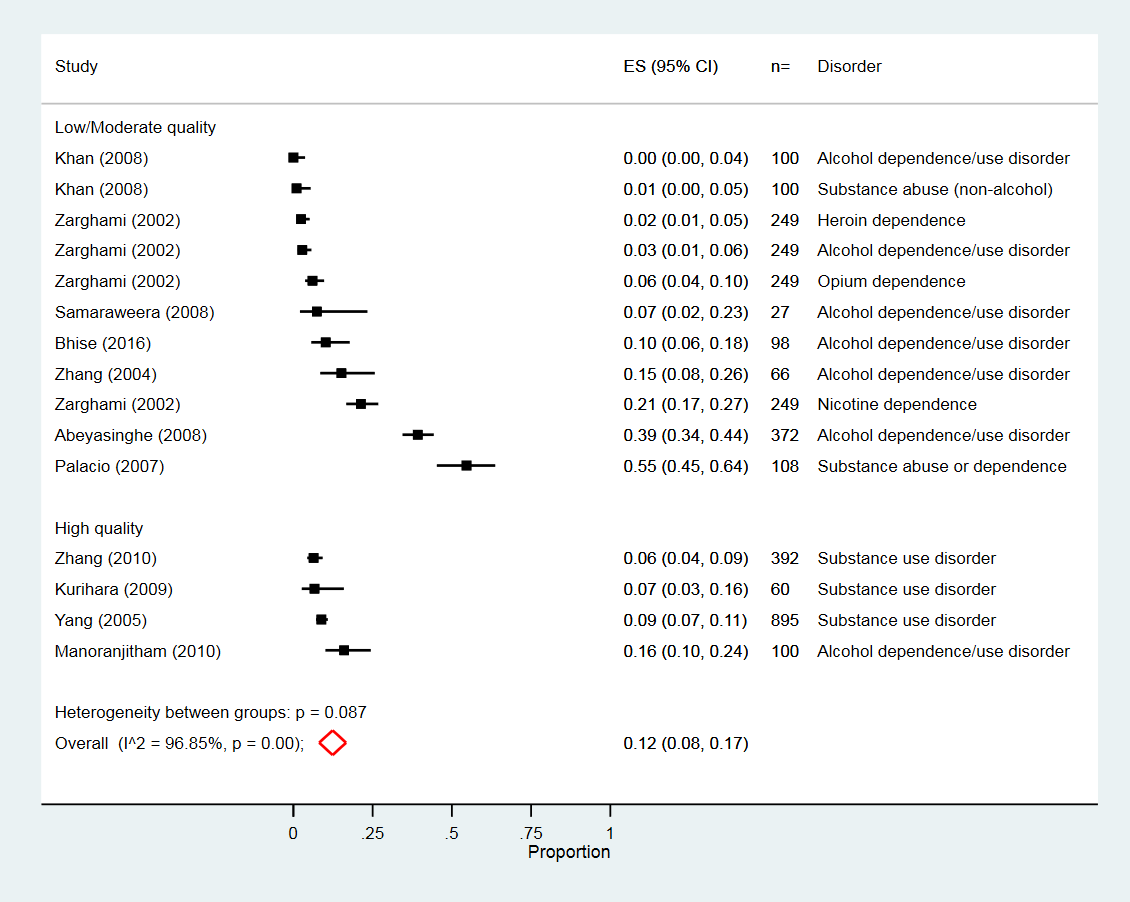
**

**Supplementary fig B – Prevalence of** s**chizophrenia, schizotypal, delusional, and other non-mood psychotic disorders in high vs. low/moderate quality studies of fatal suicidal behaviour**

**
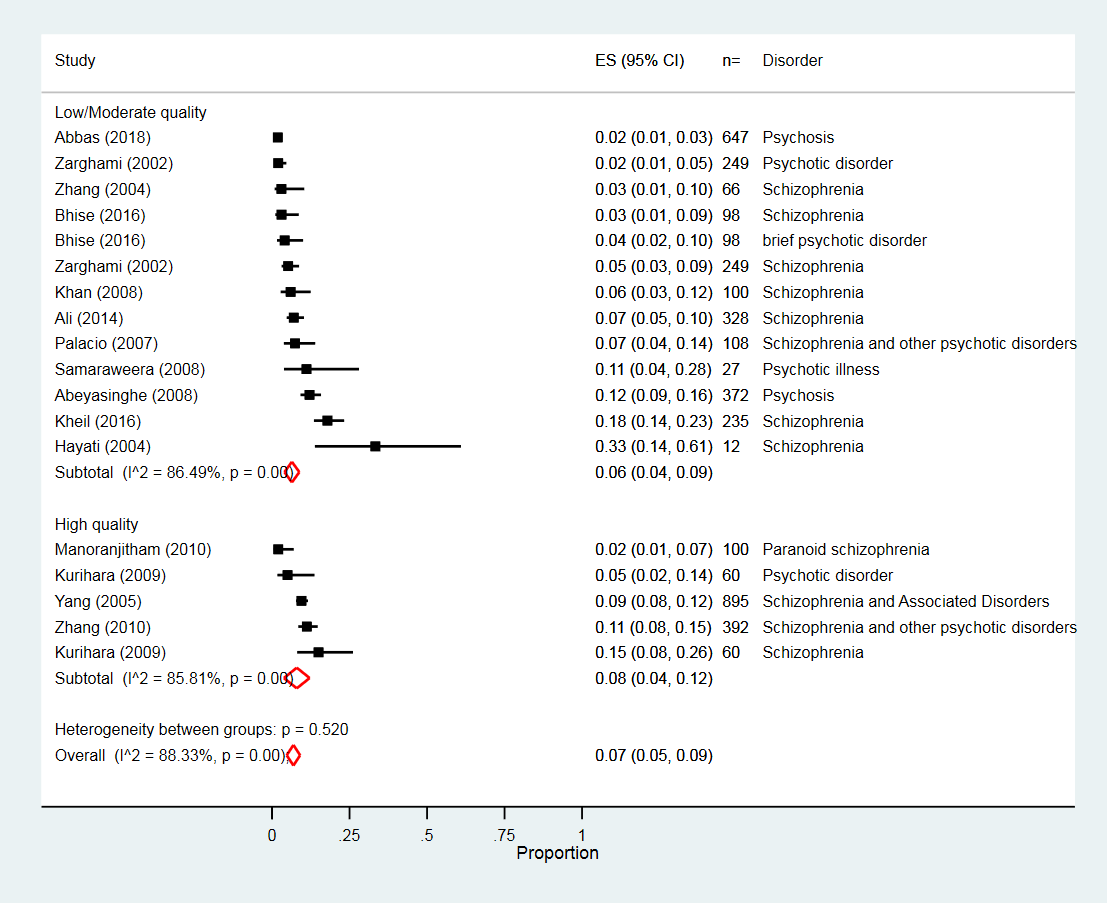
**

**
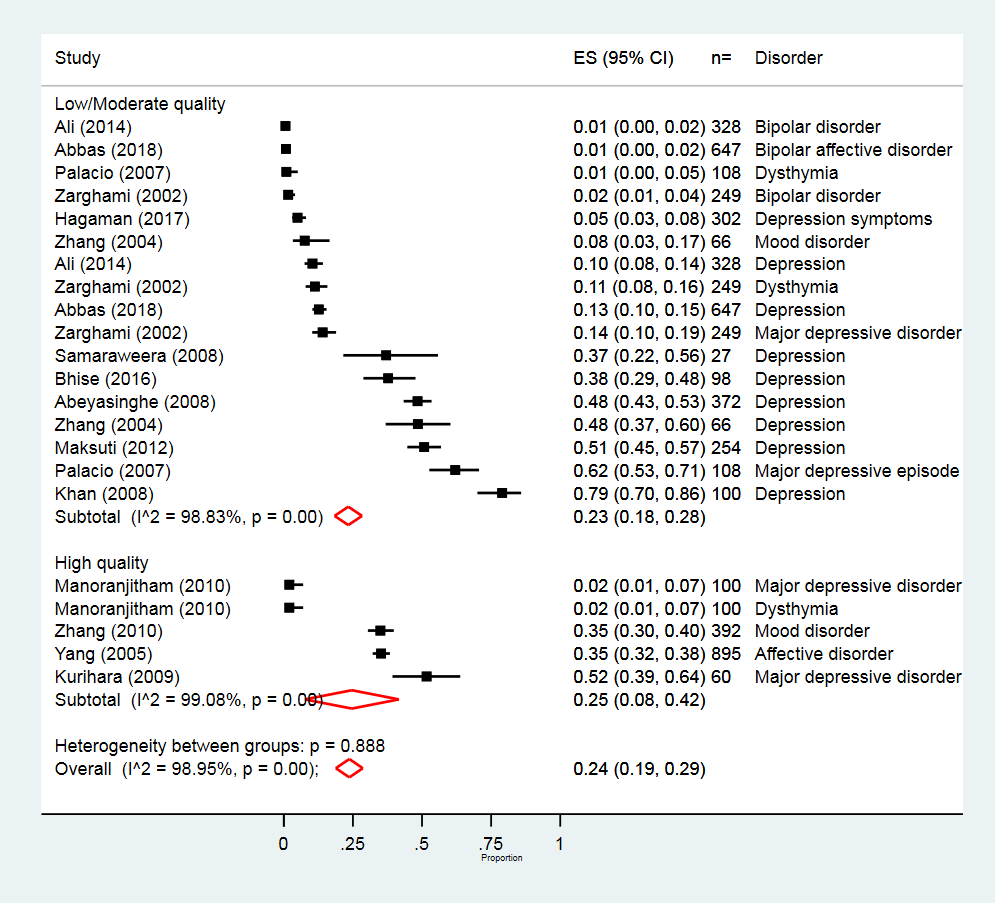
Supplementary fig C – Prevalence of mood disorders in high vs. low/moderate quality studies of fatal suicidal behaviour**

**
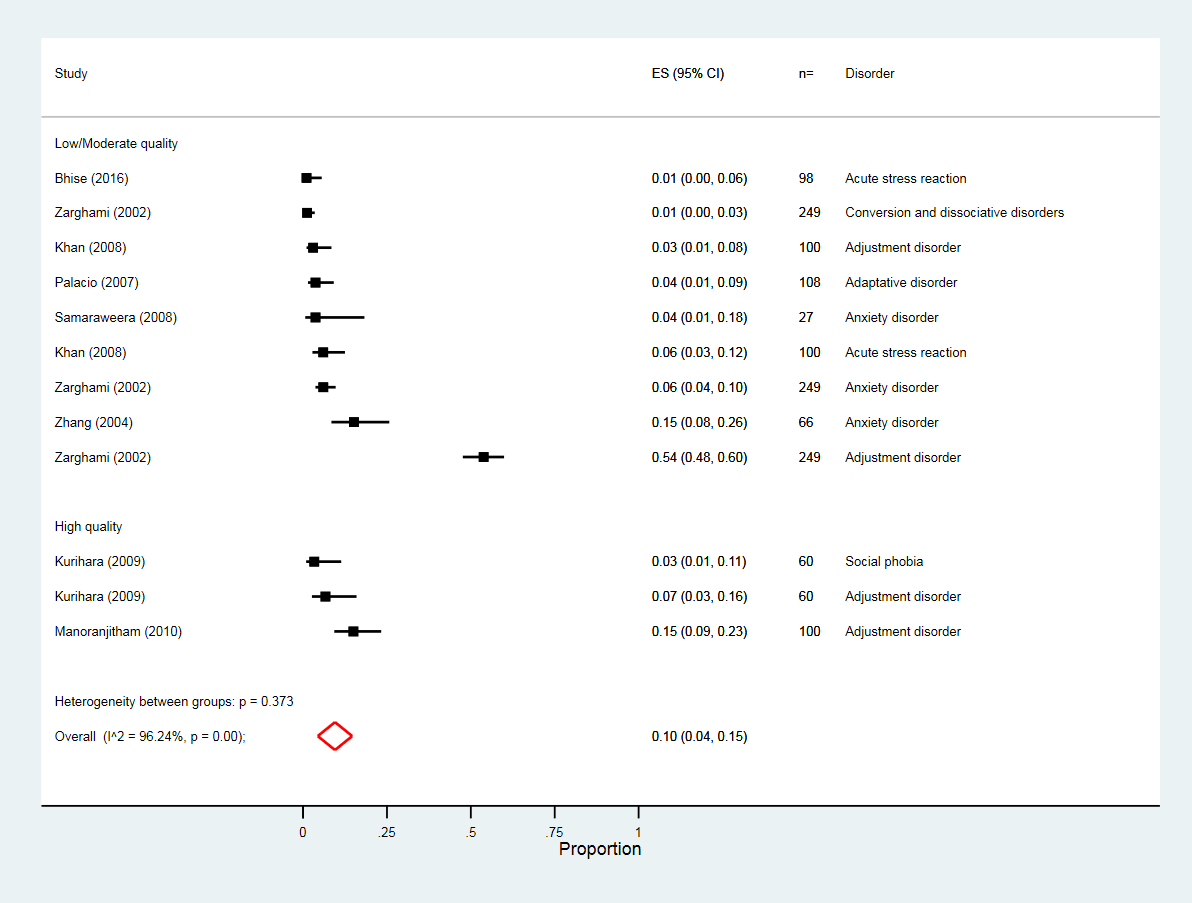
Supplementary fig D– Prevalence of anxiety, dissociative, stress-related, somatoform and other nonpsychotic mental disorders in high vs. low/moderate quality studies of fatal suicidal behaviour**

**
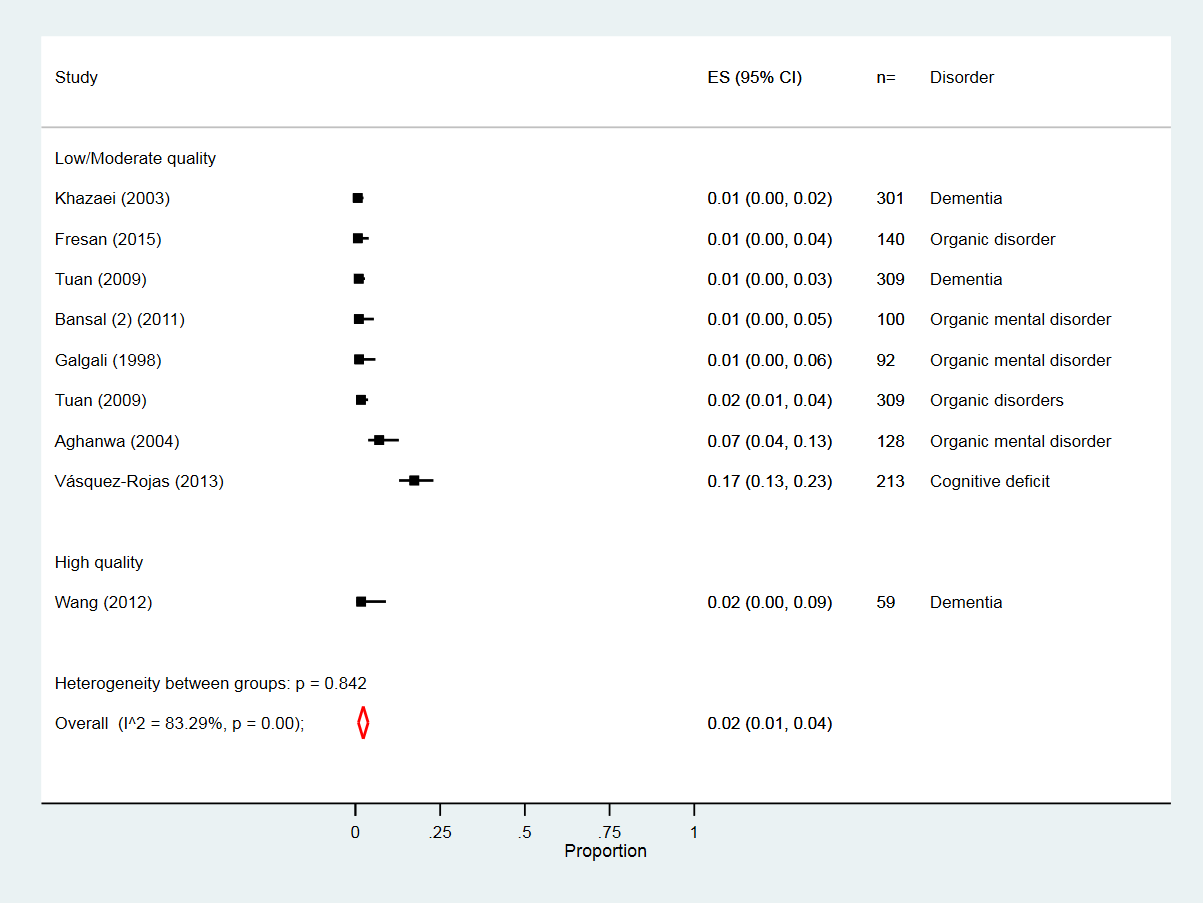
Supplementary fig E – Prevalence mental disorders due to known physiological conditions in high vs. low/moderate quality studies of non-fatal suicidal behaviour**

**Supplementary fig F – Prevalence mental and behavioural disorders due to psychoactive substance use in high vs. low/moderate quality studies of non-fatal suicidal behaviour
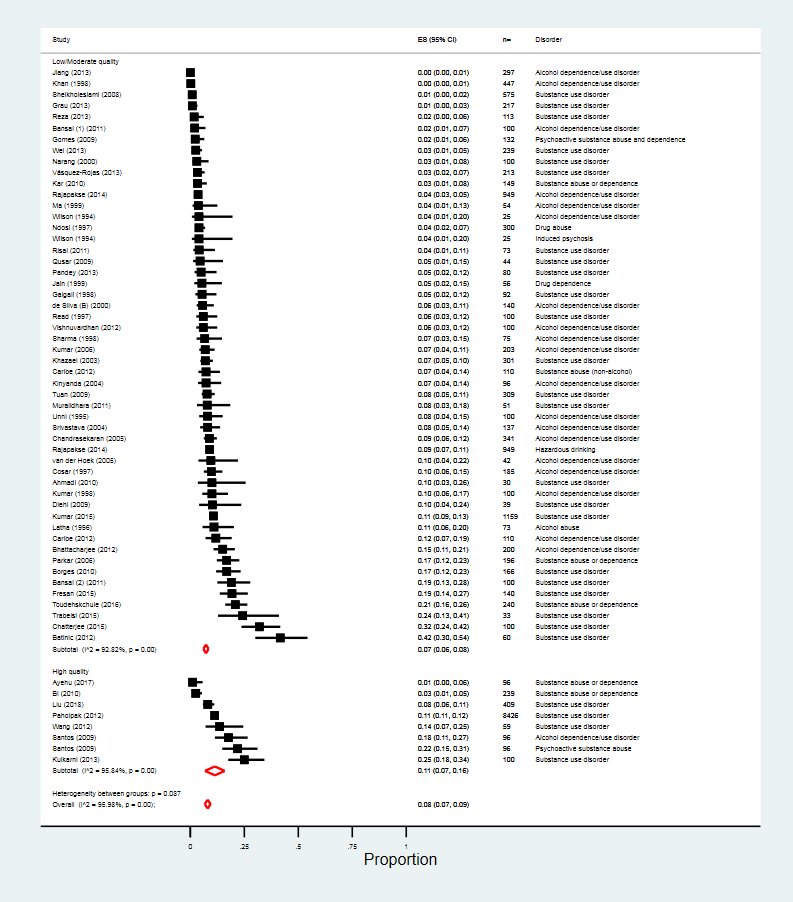
**

**Supplementary fig G – Prevalence schizophrenia, schizotypal, delusional, and other non-mood psychotic disorders in high vs. low/moderate quality studies of non-fatal suicidal behaviour**

**
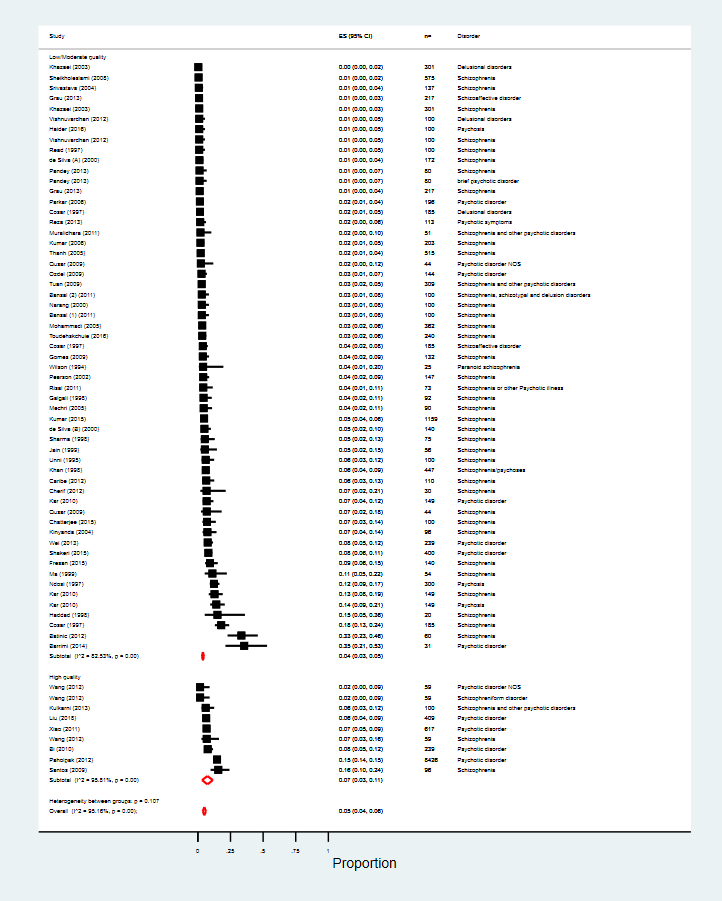
**

**
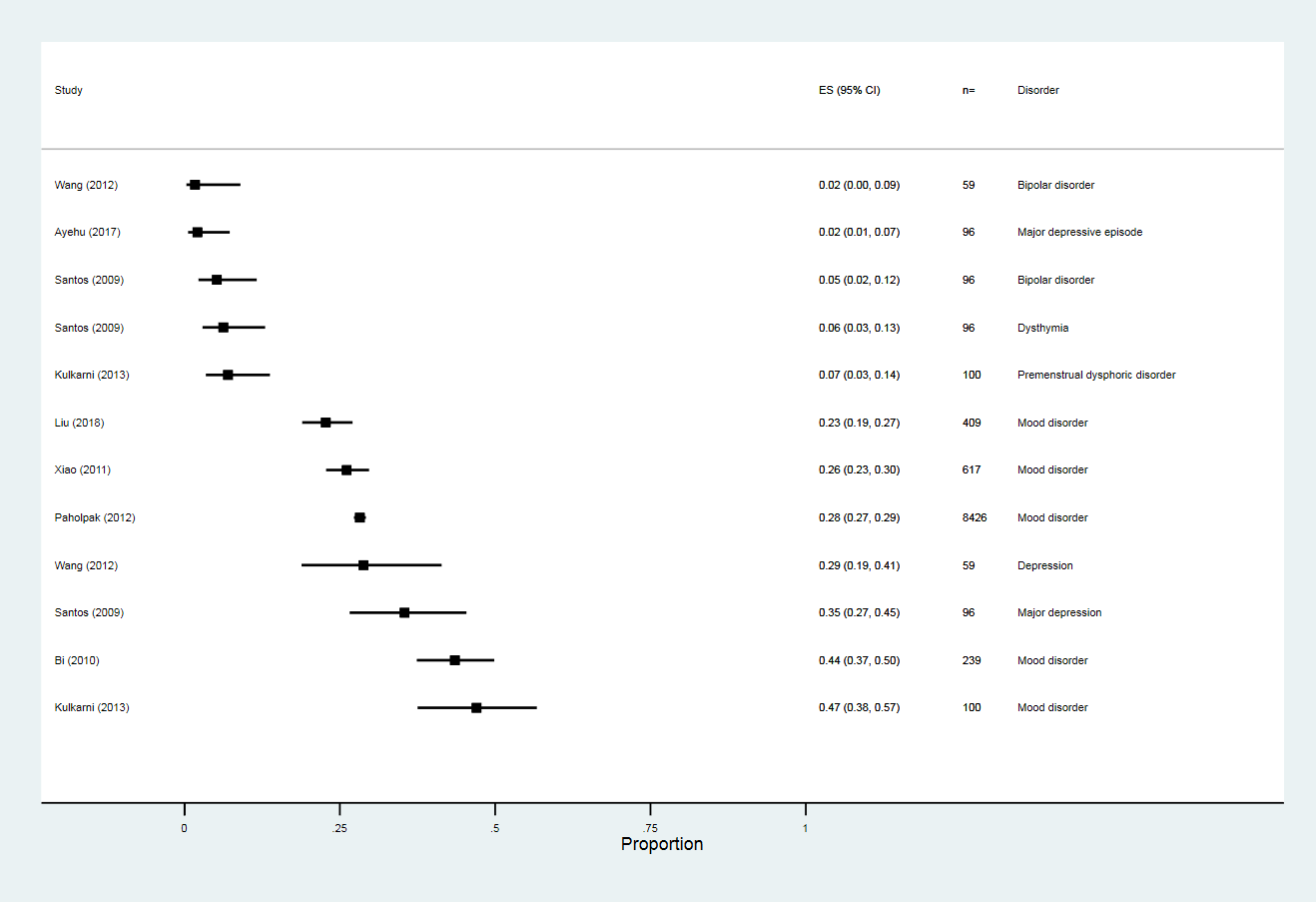
Supplementary fig H – Prevalence mood disorders in (A) high vs. (B) low/moderate quality studies of non-fatal suicidal behaviour**

**A)**

**
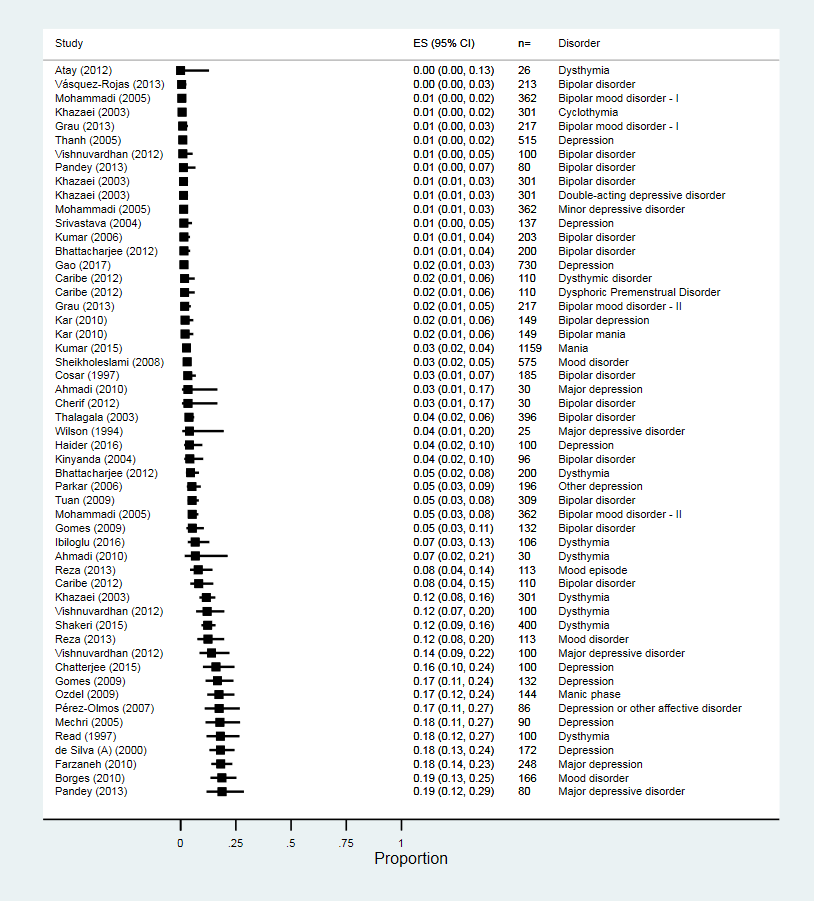
**

**B)**

**
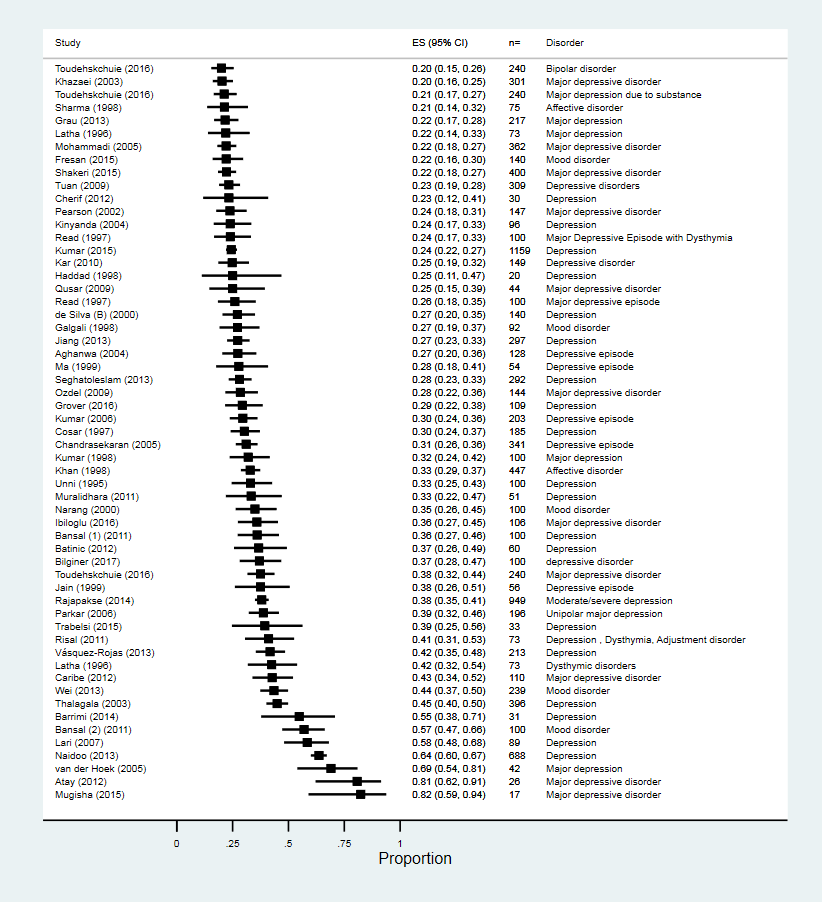
**

**B) (cont.)**

**
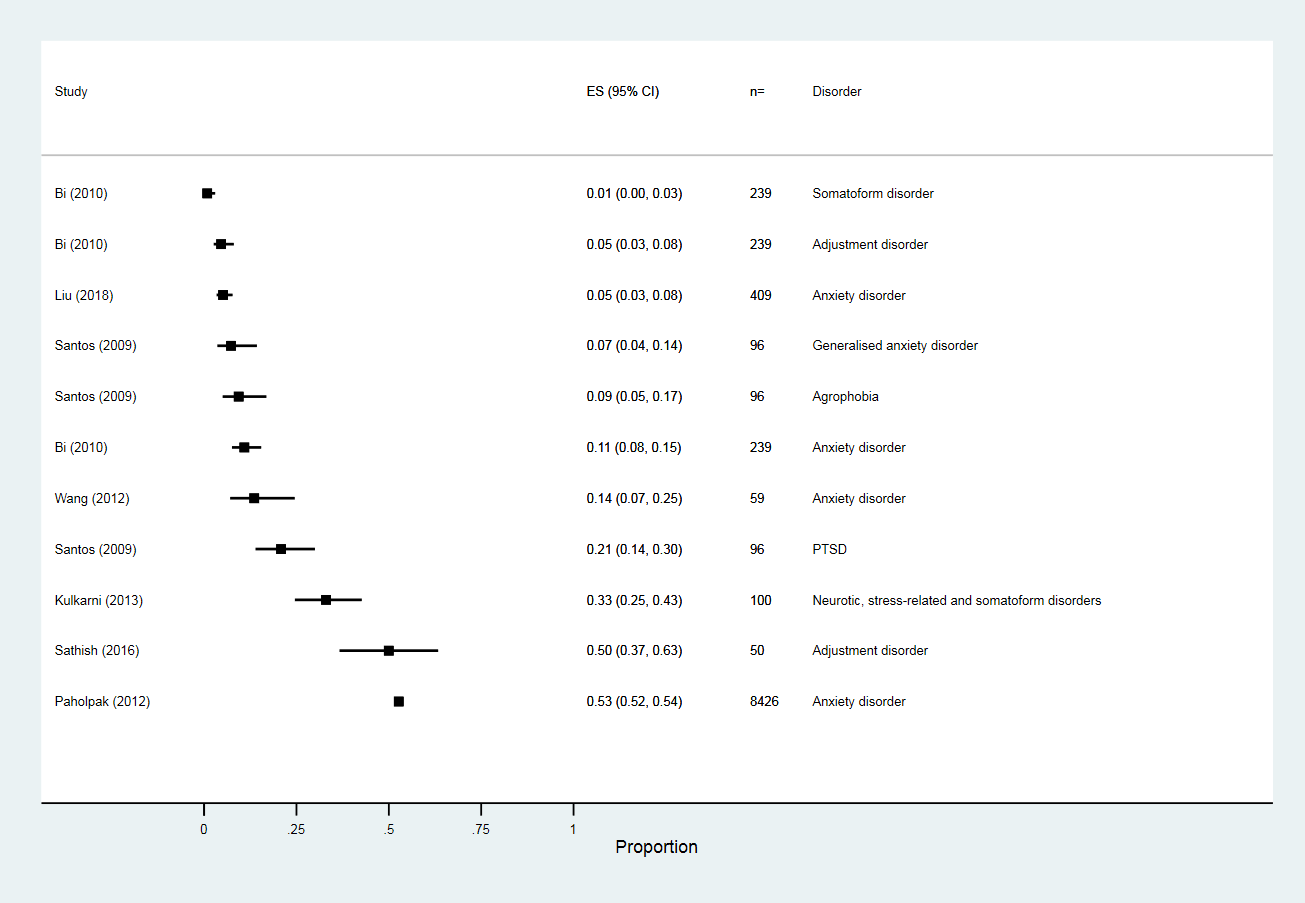
Supplementary fig I – Prevalence anxiety, dissociative, stress-related, somatoform and other nonpsychotic mental disorders in (A) high vs. (B) low/moderate quality studies of non-fatal suicidal behaviour**

**A)**

**
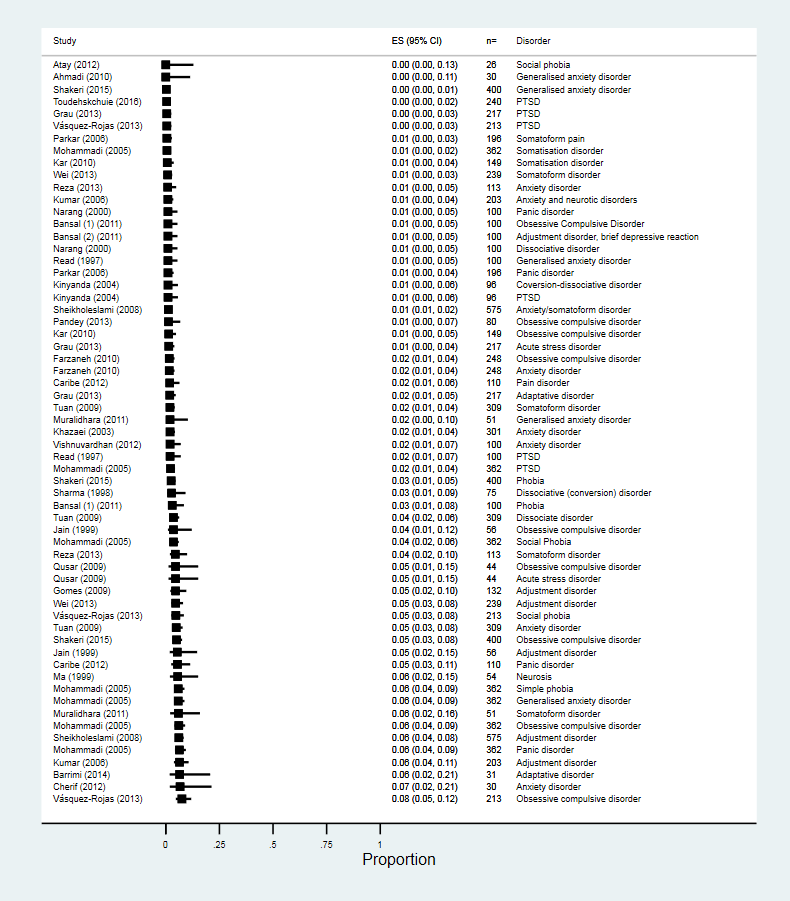
**

**B)**

**
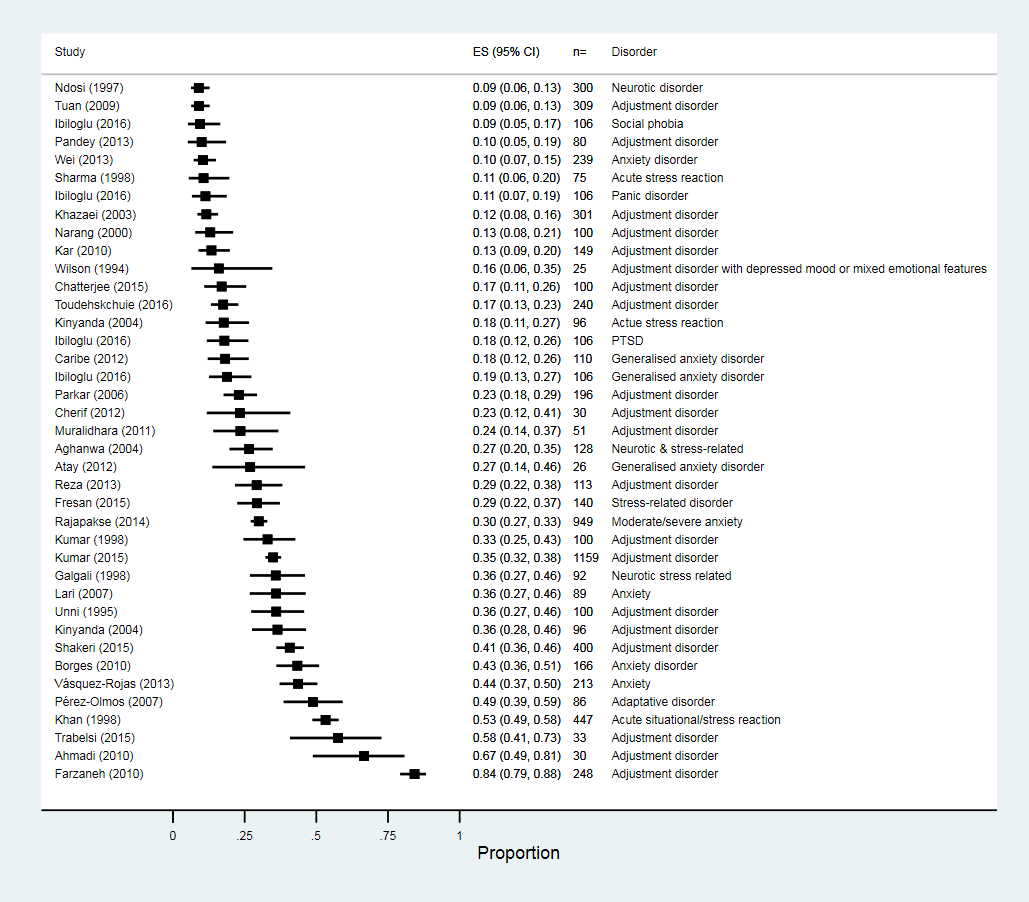
**

**B) (cont.)**

**Supplementary fig J – Prevalence of disorders of adult personality and behaviour in high vs. low/moderate quality studies of non-fatal suicidal behaviour**

**
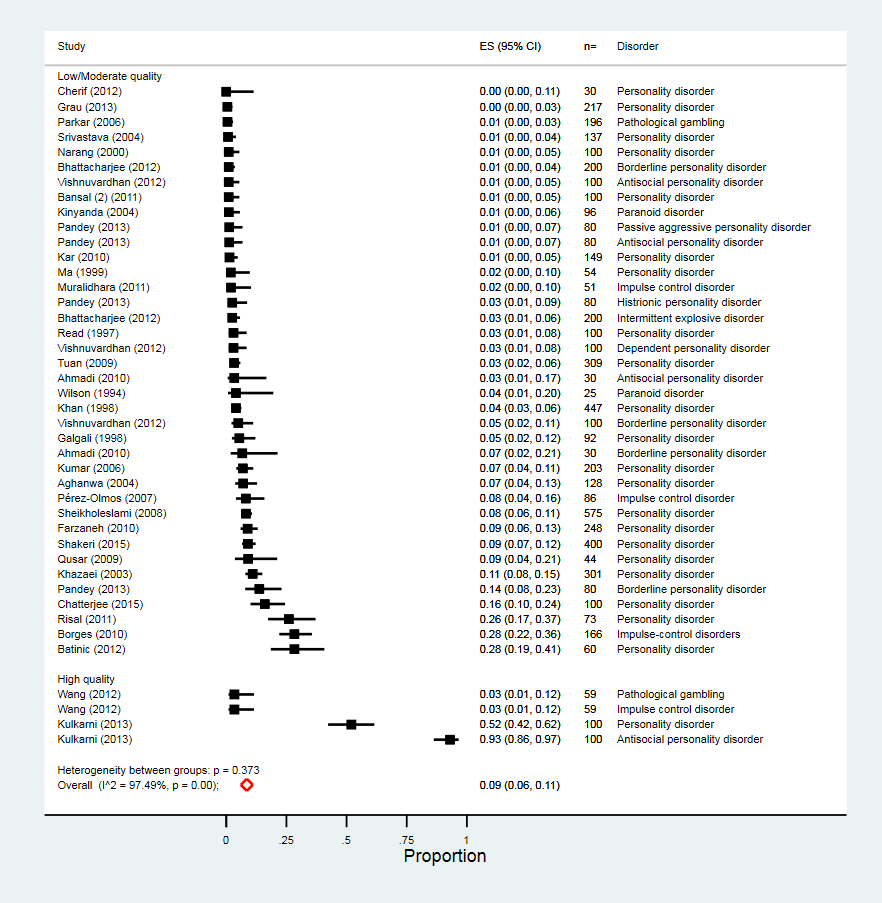
**

**Supplementary fig K – Prevalence of behavioural and emotional disorders with onset usually occurring in childhood and adolescence in low/moderate quality studies of non-fatal suicidal behaviour***

**
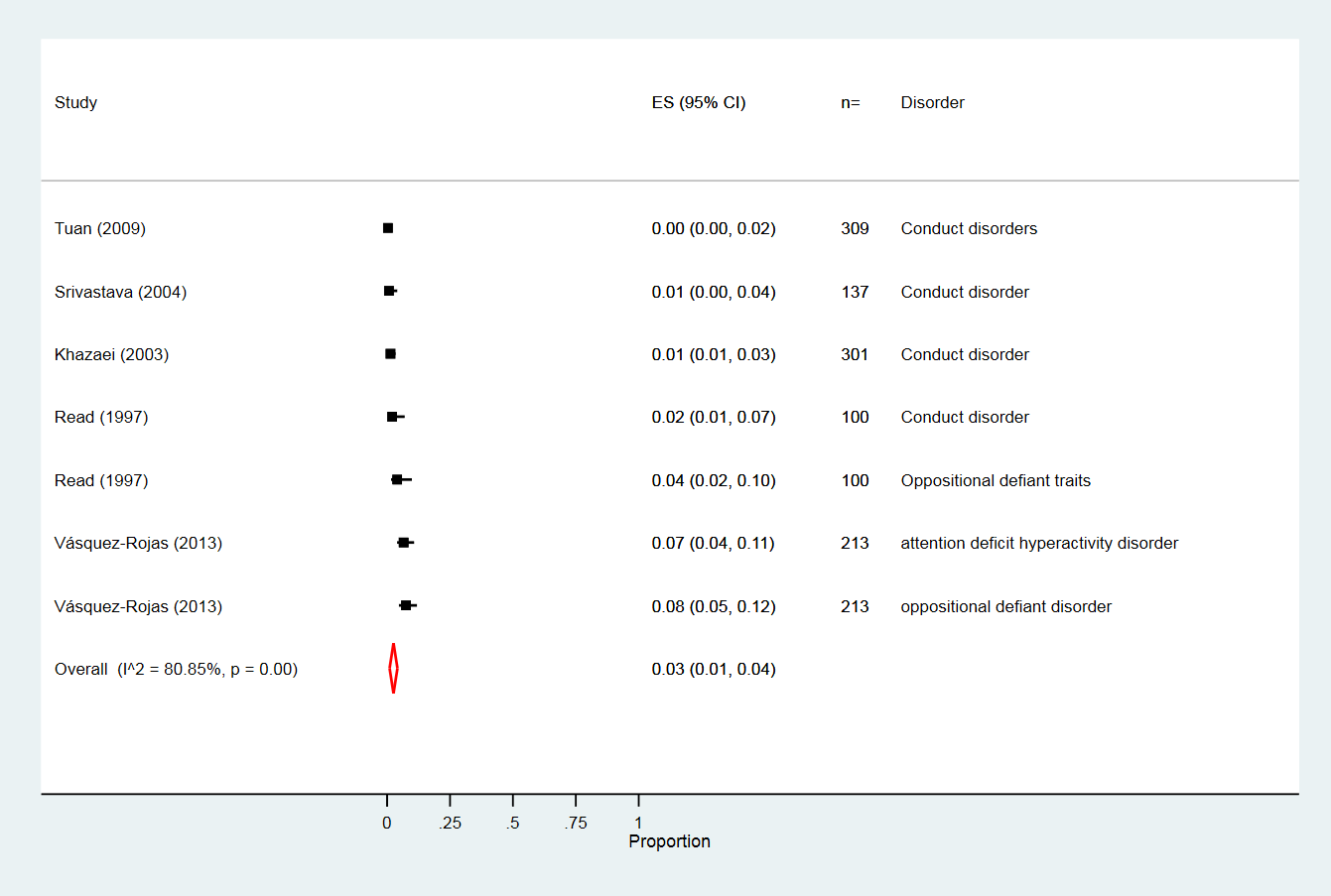
**

* no high-quality studies identified

**Supplementary fig L – Prevalence of unspecified mental disorder in fatal and non-fatal suicidal behaviour studies**

**
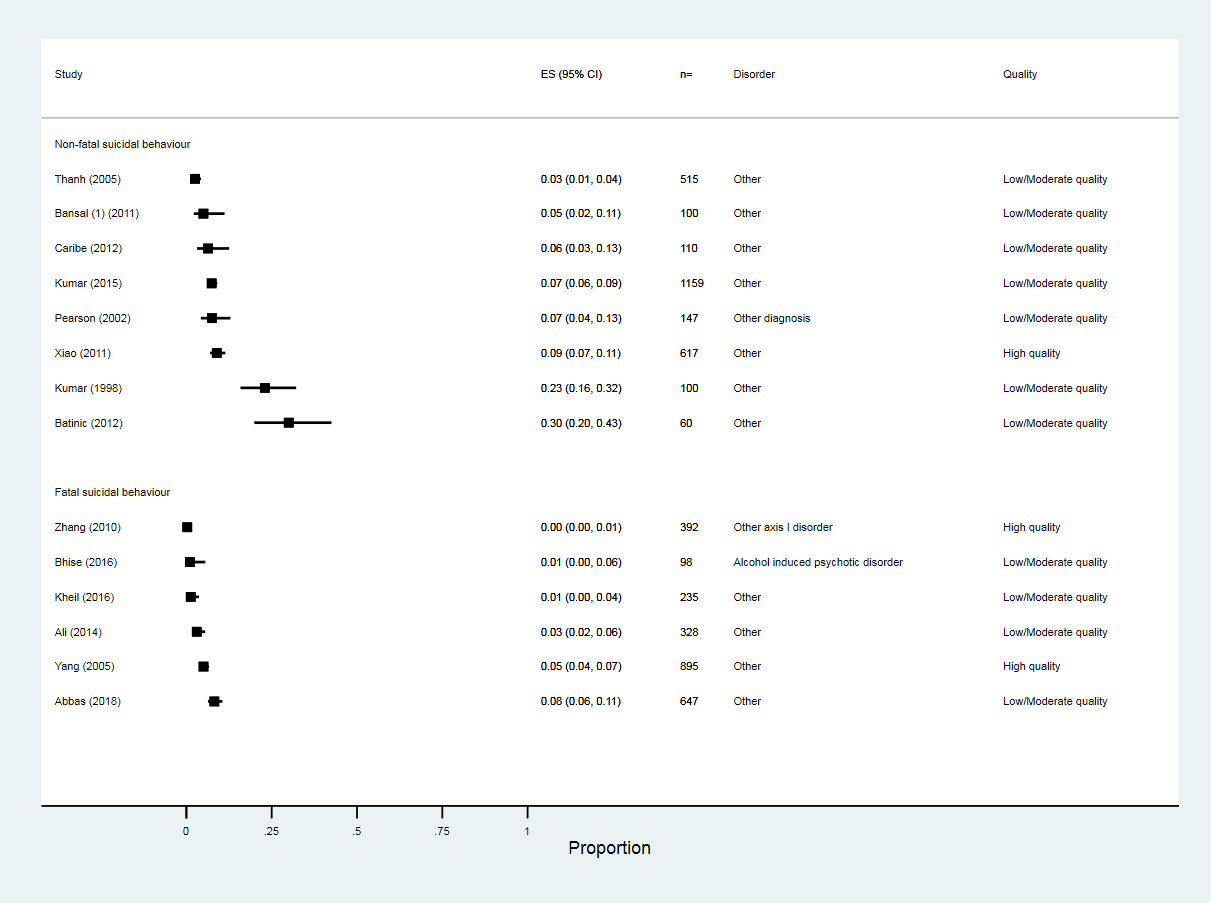
**
